# What people learn from punishment: A cognitive model

**DOI:** 10.1073/pnas.2500730122

**Published:** 2025-08-04

**Authors:** Setayesh Radkani, Joshua B. Tenenbaum, Rebecca Saxe

**Affiliations:** ^a^Department of Brain and Cognitive Sciences, Massachusetts Institute of Technology, Cambridge, MA 02139

**Keywords:** Bayesian inference, punishment, legitimacy, morality, computational modeling

## Abstract

Authorities, from parents of toddlers to leaders of formal institutions, use punishment to communicate disapproval and enforce social norms. Ideally, from whether and how severely a transgression is punished, targets and observers infer shared social norms. Yet in light of every punitive choice, observers also evaluate the motives and legitimacy of the authority. Here, we show that the effects of punishment can only be understood by considering these inferences simultaneously. We measured human observers’ joint inferences empirically in three preregistered experiments (N=1,254) and developed a rational Bayesian model using an inverse planning framework that captures and explains these inferences and their interactions quantitatively and parsimoniously. When people have different priors about norms or authorities, the model predicted and we experimentally confirmed that observing punishment by the authority can sustain polarization. This work reveals the rational logic behind how people learn from punishment and a key constraint on the function of punishment in establishing shared social norms.

## Significance

Punishment can sometimes teach and enforce social norms, but other times backfires and undermines the authority’s legitimacy. These seemingly contradictory effects of punishment can only be understood by considering the cognitive processes in the minds of human observers of punishment. In online experiments, we measured what people learn from observing punishment. We built a formal cognitive model that precisely captures and explains these inferences. Our results show that polarized interpretations of punishment arise rationally from the cognitive mechanism by which people make sense of each other’s actions. Our work illuminates a central tension faced by any authority, from institutional leaders to parents of toddlers: How to communicate social norms to some people without losing legitimacy in the eyes of others.

In the spring of 2024, university students organized widespread protests on their campuses. Consider any university where the protesting students were dispersed and suspended. Some observers of this reaction inferred that the leaders’ response was proportional and just, reinforcing the leaders’ legitimate authority, and communicating that the protests violated norms of safe and respectful campus behavior. Other observers of the same situation inferred that the leaders’ response was disproportionate and harsh, reflecting bias against the students’ position; these observers did not infer from the punishment that the protest activities violated campus norms. Equally divergent interpretations arose when leaders chose not to react punitively to the protests. The university leaders’ predicament is not unique. Structurally similar polarized interpretations of punishment arise in the context of civil disobedience, mass incarceration, and defunding police (1, 2). Indeed, on a miniature scale, similar logic arises in evaluating the punishment of a child who is disruptive at a family dinner. Here, we investigate how polarized interpretations of the same punishment arise rationally from the cognitive mechanism by which people make sense of each other’s actions.

Punishment is a cost imposed on a target, in response to an undesirable (i.e. wrong, harmful, norm-violating) action. Although punishment can deter actions directly by making them costly, effective punishment expresses disapproval of the action and promotes internalization of a norm against that action (3–7). Communicating norms is an efficient and effective way to change behavior: Once a norm is internalized, people are likely to comply with that norm even when the probability of being caught and punished is low (8). Indeed, in many contexts, authorities select when and how to punish pedagogically, and observers interpret the selected punishment as revealing the normative status of the act (6, 9–17). Harsher punishments are selected as responses to more severe transgressions (18–22). Thus, punishment can be informative for observers uncertain about the normative status or social desirability of an unfamiliar action (23, 24).

Yet choosing to punish (or not), like any social action, inevitably reveals information about the authority’s own motives and values. In some contexts, punishing creates a desirable reputation (25–28). People who incur a personal cost to punish a stranger for transgressing, in economic games or in hypothetical vignettes, are judged as more trustworthy and less selfish (29, 30), more competent and more moral (31–35), and may be more likely to be chosen as cooperation partners than people who choose not to punish the same transgression (36). Ethnographic studies confirm that third parties can gain status from punishing transgressors (37).

In other contexts, however, punishing can backfire by implying unsavory motivations of the authority, who may be perceived as less trustworthy and more selfish than those who choose not to punish (21, 27, 38–43). At the same time, punishment can fail to communicate the intended norms (44). People who observe an act being punished may not change their views of that act (17, 45–47). Indeed, harsh or selfish punishment can undermine punishment’s norm-supporting function, and even increase the prevalence of the punished behavior (48–51).

Here, we capture this range of often contradictory inferences within a single experimental paradigm by manipulating the punitive context. We build and validate the first cognitive model that parsimoniously explains when and why punishment has these specific effects on beliefs about norms and the authority’s motivations, by incorporating context as part of the phenomenon to be explained. Thus our model delineates the conditions under which an authority making punitive choices, from university leaders to parents of toddlers, could communicate moral disapproval of transgressions, without losing legitimacy and devolving into accusations of bias.

## A Formal Cognitive Model

Existing formal models characterize punitive decision-making either as a signal of the authority’s traits (e.g. trustworthiness) (36, 52) or as a means to communicate the norm that has been violated (53). These models only make heuristic assumptions about the associations between punishment and subsequent responses by the observers (e.g., ref. 36). Characterizing the cognitive processes underlying human observers’ inferences has not been the focus of previous formal models of punishment (although see ref. 13) and is indeed not accessible to traditional formal approaches to modeling social behavior (e.g., evolutionary game theory, social psychological box-and-arrow diagrams). We argue that the key to capturing the variability in what people learn from punishment is to characterize how observers of punishment update their beliefs about norms and the authority simultaneously, given the punitive context. We used cutting-edge techniques in modeling social cognition to build and test the first cognitive model of people’s moral inferences from punishment.

Our formal model is based on a generic cognitive framework for modeling observers’ inferences from others’ actions known as inverse planning (54). In this framework, observers have an intuitive generative model of how people plan actions to achieve their desires given their beliefs. By inverting this generative model, people infer the unobservable beliefs and desires that most likely generated observed actions (54–56). In this section, we describe the model structure. For full mathematical formulations, see *Materials and Methods*.

**Fig. 1.**
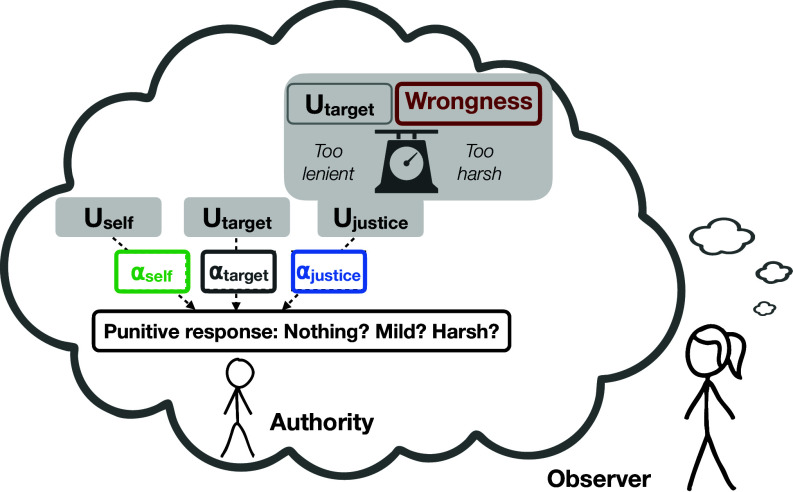
Inverse planning model, whereby observers simultaneously update their beliefs about the wrongness of the target act and the authority’s motives by performing Bayesian inference over an intuitive theory of the authority’s punitive decision-making. The authority is modeled as weighing three consequences of each possible punitive response: 1) cost and benefits for themself, 2) consequences (i.e., harm) imposed on the target, and 3) the social consequences, here instantiated as restoring justice by balancing the negative consequences imposed on the target against the wrongness of the target act (i.e., harshness). The utility terms ($U_{x}$) indicate the consequences, and the weights on the utilities ($\alpha_{x}$) indicate how much the authority values and incorporates each consequence into their decision-making (i.e., the authority’s motives). Observers use this model to update their subjective beliefs about the authority’s motives and about the wrongness of the target action (i.e. the severity of the norm violation).

We propose that people use inverse planning to interpret punishment, as a goal-directed action chosen by the authority. Observers have an intuitive generative model of how an authority with certain motives would respond to a target act with a certain level of wrongness. The generative model specifies the consequences of each possible punitive response that observers expect authorities to consider when making their choice (Fig. 1): i) the direct costs and benefits to the self, ii) the cost imposed on the target of punishment, and iii) the proportionality of the response to the violation of norms. We call these consequences “utilities,” because they can be weighed, summed, and compared, to generate choices (56). Observers use the authority’s choice to infer the weight they placed on each type of consequence. Thus, the model operationalizes the psychology of legitimacy (8). To be perceived as legitimate, authorities must be seen as unselfish, impartial, and benevolent (57–59).

First, the authority considers the direct costs and benefits to themself of each possible action ($U_{\text{self}}$. In prior theorizing, punishment is often conceptualized as a form of altruism (e.g., refs. 60 and 61): potentially beneficial to the group but costly to the individual (in terms of fitness costs, or risks of retaliation). Yet punishers may directly benefit from their punishment (e.g., when the authority keeps the material gains or is rewarded for punishment; ref. 39). Thus, we allow possible punishments to range from directly costly to directly beneficial to the authority. Each authority assigns different weights to these consequences. The more selfish the authority (higher $\alpha_{\text{self}}$), the more direct costs or benefits will influence their punitive choice. Costly punishment is more likely to be chosen by an unselfish authority (lower $\alpha_{\text{self}}$).

Second, by definition, punishment imposes a direct cost on its target ($U_{\text{target}}$, always negative). A biased authority may have an independent motive to harm (negative $\alpha_{\text{target}}$) or protect (positive $\alpha_{\text{target}}$) the target of punishment. People sometimes select harsher punishment for competitors or outgroup members, and arrange lenient punishment for cronies (40, 62–64). Impartial authorities (small $\alpha_{\text{target}}$) are not influenced by personal relationships with the target in their punitive choice (65).

Third, the authority considers the social benefit of punishment, here expressed as proportionality of the punitive response to the wrongness of the transgression ($U_{\text{justice}}$). Proportional punishment may be directly desirable (e.g., as retribution, “an eye for an eye,” 66, 67), or may be desired as a means to restore social balance (68), or create efficient deterrence (69). An authority who cares a lot about proportionality ($\alpha_{\text{justice}}$) would choose mild punishment for moderately serious transgressions and harsh punishment for very serious ones, whereas an authority who does not particularly care about justice would be relatively indiscriminate. Conceptualizing the social good of punishment in terms of proportionality proves critical to capturing the communicative function of punishment, as it makes the punitive choice informative about the degrees of wrongness or moral disapproval of the punished transgression (5, 12, 17, 24).

The observer can use this mental model to interpret the authority’s punitive decisions. That is, from observing whether and how severely an authority punishes an act, the observer can simultaneously and rationally update their beliefs about four unobservable parameters in their causal model: the authority’s selfishness ($\alpha_{\text{self}}$), bias ($\alpha_{\text{target}}$), and justice motive ($\alpha_{\text{justice}}$), as well as the wrongness of the target act (wrongness). Note that these four parameters express the observer’s subjective beliefs about the authority’s motives and the wrongness of the target act, and thus need not to be true. Similarly, the generative model need not be a correct or complete model of the authority’s actual planning process; it only has to capture the planning process as imagined by the observers (55).

A key distinctive prediction of our inverse planning model is that parameter updates from observed punishment are interdependent: Each belief update depends on both the value and the confidence of the observers’ prior beliefs about all the other parameters. We test this prediction across three studies, by experimentally manipulating the observers’ priors (through manipulating the punitive context) and measuring their inferences. Then, we compare this full model to non-Bayesian control models inspired by the existing literature that assume heuristic associations between features of punishment (i.e., cost to the target, cost or benefit to the punisher) and what observers learn about either the social norms or the authority (30, 36, 39, 52, 53).

## A Paradigm for Examining Joint Inferences from Punishment

**Fig. 2.**
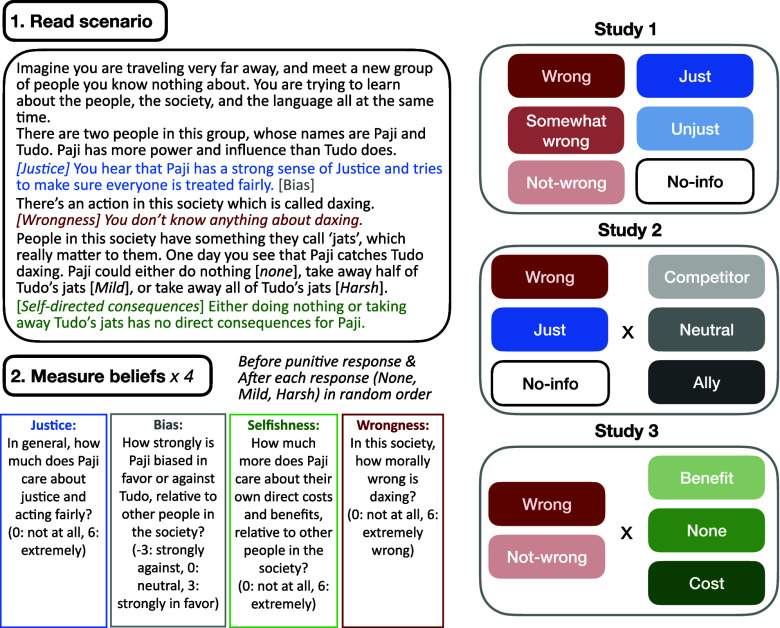
Experimental design and prior conditions in studies 1 to 3, for one example scenario and condition. Six base scenarios were written in the same structure as this example; across the scenarios, we varied the name of the authority and the target, the target act, the means of punishment, and the self-directed consequences for the authority (wherever applicable). All questions were measured on a 7-point Likert scale, and participants indicated their response using a slider or chose “I don’t know. All values are equally likely.” The colored information was manipulated depending on the prior condition (*Materials and Methods* and *SI Appendix*). The text in the brackets did not appear in the scenario. There were six prior conditions in study 1, 9 (in a 3 × 3 design) in study 2, and 6 (in a 2 × 3 design) in study 3.

To ensure experimental control over people’s prior beliefs, we developed six scenarios in hypothetical societies with unfamiliar characters, actions, and punishments. Each scenario introduced two characters (an authority and a target), a novel target action, and a novel means of punishment. The authority catches the target doing the target action (e.g., “daxing”), and then the authority chooses from three possible responses that harm the target severely, mildly, or not at all (e.g., “take away all/half/none of Tudo’s jats”). Across our scenarios, punitive responses implied loss of goods, loss of privilege, imposition of an unpleasant task, and so on, to increase the generalizability of our findings (*SI Appendix*, Fig. S1). We experimentally manipulated participants’ priors about the wrongness of the target action (studies 1 to 3), the authority’s justice motive (studies 1 to 3), the authority’s bias toward the target (study 2), and the direct consequences of punishment for the authority (study 3). Each participant answered four questions, four times, for each scenario. We measured prior beliefs about the wrongness of the target act and the motives of the authority (i.e., justice, bias, and selfishness) before the authority chose a response. We then measured each participant’s updated beliefs after learning that the authority chose each of the three possible punitive responses [the “strategy method” (36, 70), order randomized within scenarios and participants]. For each punitive response, participants also reported the harshness of the response, the cost for the target, and the cost or benefit for the authority (for full wording of dependent variables, see *Materials and Methods*). The overall design of all three studies, a template of an example scenario, and all dependent variables, are shown in Fig. 2. Participant demographics were collected after the experiment (gender, age, race, self-reported ideology, and right-wing authoritarianism). Predictions, designs, and analysis plans for all three studies were preregistered simultaneously prior to data collection (https://osf.io/hjcrz). Any deviation from the preregistrations is marked with (*NP).

## Inferences About Wrongness and the Authority’s Motives Are Interdependent (Study 1)

In the first experiment, we independently manipulated prior beliefs about either wrongness or the authority’s justice motives. In a within-subject design, each participant (N=358) read 6 scenarios. Across the 6 conditions, the scenario provided prior information about only the justice motives of the authority (“Just,” “Unjust;” stating the authority cares or does not particularly care about justice and acting fairly), only the wrongness of the target act (“Wrong,” “Somewhat-wrong,” “Not-wrong;” stating the harmfulness and prevalence of the act), or neither (“No-info”) (Fig. 2; see *SI Appendix*, Fig. S2.1 for the exact wording of manipulations; *SI Appendix*, Fig. S2.3 for the Manipulation check pilot results). We stipulated that either choosing to punish or doing nothing had no direct costs or benefits for the authority and provided no information about the authority-target relationship. A pilot version of this experiment, with five conditions (all except for “No-info”), independent participants, and highly similar results, was reported in ref. 71.

### Experimental Results.

Consistent with the communicative function of punishment, overall, participants inferred that punished actions were more wrong than not-punished actions (punish vs. not-punish: β=0.649, std = 0.044, t(357) = 14.70, P< 2e-16). However, the efficacy of punishment in communicating wrongness depended on observers’ prior beliefs about both wrongness and the authority’s motives.

When observers were uncertain about wrongness but believed the authority cares about justice (i.e., “Just” condition), beliefs about wrongness changed substantially as a result of the authority’s punitive decision (punish vs. not-punish: β=1.281, std = 0.095, t(714) = 13.52, P< 2e-16) and its severity (harsh vs. mild: β=0.607, std = 0.103, t(5.1) = 5.88, P=0.0019). However, punishment failed to communicate wrongness if the authority was described as not particularly concerned with justice (interaction between response (punish vs. not-punish) and condition (“Unjust” vs. “Just”): β=−0.586, std = 0.112, t(35.2) = −5.22, P= 8.26e-06; interaction between response (harsh vs. mild) and condition: β=−0.291, std = 0.114, t(9.8) = −2.55, P=0.0292).

**Fig. 3.**
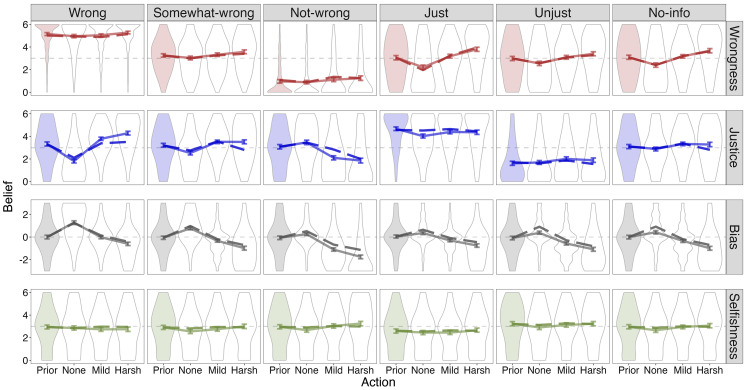
Study 1 results, across all 6 scenarios. Each row corresponds to a dependent variable, and each column to a prior condition. Each panel (N=358) shows the mean and the distribution of prior beliefs (shaded distributions) and posterior beliefs (empty distributions) after learning that the authority chose to do nothing, punish mildly, or punish harshly. The solid lines connect the mean of human judgments (with bootstrapped 95% CI of the mean as error bars), averaged across 6 scenarios. The dashed lines connect the predictions of the best-fitting inverse planning model on the held-out scenarios, averaged across the 6 scenarios.

When participants had strong prior beliefs about the wrongness of the target act (i.e., “Wrong,” “Somewhat-wrong,” and “Not-wrong” conditions), punishment had significantly less influence on beliefs about wrongness (Fig. 3; interaction between response (punish vs. not-punish) and condition (“Not-wrong” vs. “Just”): β=−0.997, std = 0.144, t(5.5) = −6.91, P= 6.6e-04; interaction between response (harsh vs. mild) and condition, β=−0.470, std = 0.121, t(8) = −3.88, P=0.0047), even when beliefs about the authority’s justice motives were similar (interaction between response (punish vs. not-punish) and condition (“Somewhat-wrong” vs. “No-info”): β=−0.602, std = 0.108, t(8.6) = −5.57, P= 4.1e-04; interaction between response (harsh vs. mild) and condition, β=−0.242, std = 0.099, t(23.8) = −2.44, P=0.0225).

At the same time, punishment contained information about the authority’s motives. Overall, participants inferred that an authority who chose to punish was more motivated by justice, but more biased against the target, than an authority who chose not to punish (justice: β=0.460, std = 0.064, t(11.8) = 7.10, P= 1.36e-05; bias: β=−1.311, std = 0.0697, t(24.5) = −18.81, P= 4.44e-16). However, the reputational consequences of punishment depended sensitively on observers’ priors about wrongness and the proportionality of the punitive choice.

When participants had strong prior beliefs that the target action was wrong, authorities who punished were perceived to be much more motivated by justice than authorities who did not punish (punish vs. not-punish: β=2.222, std = 0.119, t(10.3) = 18.74, P= 2.73e-09; harsh vs. mild: β=0.494, std = 0.106, t(101) = 4.65, P= 9.97e-06). However, when observers believed that the target act was not wrong, the authority who did punish was perceived as less motivated by justice (e.g., interaction between condition (“Not-wrong” vs. “Wrong”) and response (punish vs. not-punish): β=−3.704, std = 0.236, t(5.1) = −15.68, P= 1.65e-05; response (harsh vs. mild): β=−0.737, std = 0.151, t(21.7) = −4.87, P= 7.54e-05); indeed, this inference varied continuously with wrongness and punitive harshness (e.g., interaction between condition (“Somewhat-wrong” vs. “Wrong”) and response (punish vs. not-punish): β=−1.237, std = 0.171, t(7.1) = −7.22, P= 1.61e-04; (harsh vs. mild): β=−0.502, std = 0.181, t(7.7) = −2.78, P=0.0248).

Similarly, the authority was judged to be highly biased against the target after punishing not-wrong actions (compared to wrong actions, β=−1.067, std = 0.084, t(357) = −12.74, P< 2e-16), and highly biased in favor of the target after doing nothing in response to wrong actions (compared to not-wrong actions, β=0.975, std = 0.091, t(357) = 10.69, P< 2e-16).

Authorities who punished were perceived to be slightly more selfish than those who did not (punish vs. not-punish: β=0.234, std = 0.045, t(62.4) = 5.16, P= 2.77e-06), and these judgments also varied based on wrongness. For further analyses, see *SI Appendix* for study 1.

### Computational Model Fits.

We simulated the inverse planning model to assess its performance in capturing belief updates after observing each authority’s response, within each scenario and prior condition. The inverse planning model, fit to study 1 data, captured all the nuanced effects of prior beliefs on the observers’ belief updates (Fig. 3, dashed line). That is, the inverse planning model was able to predict, within a single framework, the full range of inferences about wrongness and the authority’s motives, depending on the value and uncertainty of prior beliefs about both.

To estimate the accuracy of the inverse planning model, we used cross-validation: We iteratively held out all six conditions of one scenario as the test data and found the best-fitting parameters to capture four dependent variables simultaneously on all six conditions in the five remaining scenarios. These parameters were then used to predict inferences for all variables in all conditions in the held-out scenario, and model performance was assessed by measuring Pearson’s correlation (r). We report the average cross-validated r across six held-out scenarios as the final performance measure.

Next, we compared the inverse planning model to two sets of control models. The inverse planning model uses a single set of parameters to simultaneously predict all four dependent variables. The control models operationalize the separation, in existing models of punishment, between inferences about the target act (13, 53) vs. the authority (36, 52). Each set of control models consists of four separate linear regressions, each predicting belief updates for one dependent variable. The first set of models use the harm imposed on the target ($U_{\text{target}}$) as the predictor, while the second set uses both the harm imposed on the target and the direct consequences of the response for the authority ($U_{\text{self}}$). We compared the performance of the inverse planning model vs. the control models, using both average cross-validated Pearson Correlation (r) and bootstrapping (for details of model fitting and comparison, see *Materials and Methods* and *SI Appendix*).

**Fig. 4.**
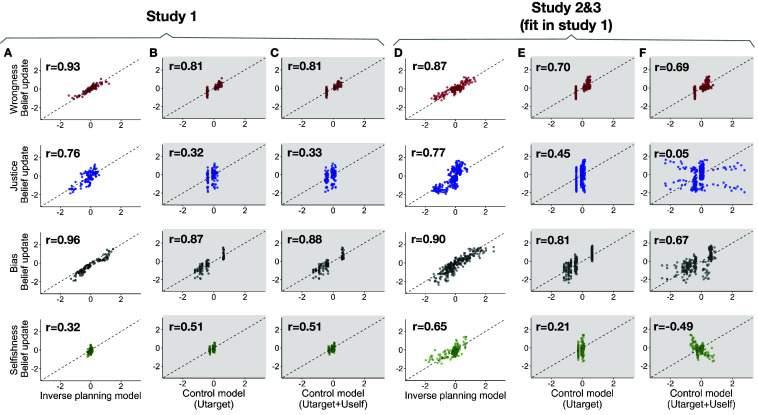
Performance of the inverse planning and control models in capturing observers’ judgments in study 1 (*A*–*C*) and studies 2 and 3 combined (*D*–*F*). In each plot, the y-axis shows human belief updates (i.e., posterior minus prior belief), and x-axis shows model predictions for the belief updates. Each data point corresponds to one punitive response in one scenario and prior condition (e.g., in study 1, sample average of belief updates after learning that the authority chose to do nothing in scenario 1 in the “Wrong” condition); therefore, each plot contains 3 responses × 6 scenarios × N prior conditions data points, where N=6 for studies 1 and 3, and N=9 for study 2. In study 1, the model used to make predictions for each scenario is fit only on the other 5 scenarios. In studies 2 and 3, the model used to make predictions for all scenarios is fit only on the data from study 1.

The joint inference model explained substantial variance in posterior beliefs for all variables simultaneously (Fig. 3, dashed line; average r on the held-out scenario = 0.99, 0.90, 0.96, 0.63 for wrongness, justice, bias, and selfishness, respectively). As a stricter test, the model explained substantial variance in the belief updates specifically (Fig. 4*A*, average r on held-out scenario = 0.93, 0.76, 0.96, 0.32). Note that the lower explained variance of selfishness judgments reflects the design of this experiment: Punishing and not punishing were stipulated to have no direct costs or benefits for the authority, so the model’s belief updates were small and noisy (in contrast to Study 3, below).

Although the control models were optimized to capture each dependent variable separately, they all performed worse than the inverse planning model in capturing observers’ inferences in Study 1 (Fig. 4 *B* and *C*; model comparison using bootstrapping in *SI Appendix*, Fig. S2.8 with all ps < 0.001, except for selfishness judgments). Even when the empirically estimated prior beliefs for each condition, for the same variable, were added as additional regressors to the control models, the inverse planning model still outperformed the control models (*SI Appendix*, Figs. S2.7 and S2.8), highlighting the importance of considering the interaction between beliefs about wrongness and various motives of the authority.

## Generalization to Novel Contexts (Studies 2 and 3)

Next, we tested how well our model could generalize to novel contexts by manipulating additional features of the punitive context compared to study 1. Across two studies, we replicated our key findings in study 1 in independent datasets and further tested the effects of varying prior beliefs about the authority’s bias (study 2; N=535) and the costs or benefits of punishment for the authority (study 3; N=361).

Both studies included a subset of prior conditions from study 1, crossed with manipulation of beliefs about other key variables in the model. In study 2, prior information about wrongness (i.e., “Wrong” condition), authority’s care for justice (i.e., “Just” condition), or neither (i.e., “No-info” condition) was crossed with three levels of prior belief about the authority’s bias, by manipulating the authority-target relationship (Fig. 2; “Ally,” “Neutral,” “Competitor;” *SI Appendix*, Fig. S3.1). The “Neutral” conditions directly replicated study 1. An authority who is an “Ally” with the target was judged to be biased in favor of the target, whereas a “Competitor” authority was judged to be biased against the target (Manipulation check pilot, *SI Appendix*, Figs. S3.3 and S3.4). Each participant saw all three levels of allyship crossed with two (of three) prior conditions (i.e., 6 of 9 conditions), in 6 unique scenarios.

Study 3 manipulated the consequences for the authority. Prior information about wrongness (i.e., “Wrong” and “Not-wrong” conditions) was crossed with three levels of consequences of punishment for the authority (“Cost,” “No-consequence,” or “Benefit”), compared to doing nothing which was always described as having no consequences for the authority. The “No-consequence” conditions directly replicated study 1. Each participant read all 6 scenarios, one in each of the 6 conditions.

We then tested how well the inverse planning model, with parameters fit to the data in Study 1, could generalize to and quantitatively predict the inferences in Studies 2 and 3, i.e., generalizing to independent data from new participants and to new punitive contexts (Fig. 4*D*). We compared the inverse planning model to the same control models fit to study 1 data, as above (Fig. 4 *E* and *F* and *SI Appendix*, Figs. S5.1 and S5.2). Here, we report the results for all the prior conditions from studies 2 and 3 combined; for separate results of studies 2 and 3, see *SI Appendix* for each study.

### Replicating Study 1.

**Fig. 5.**
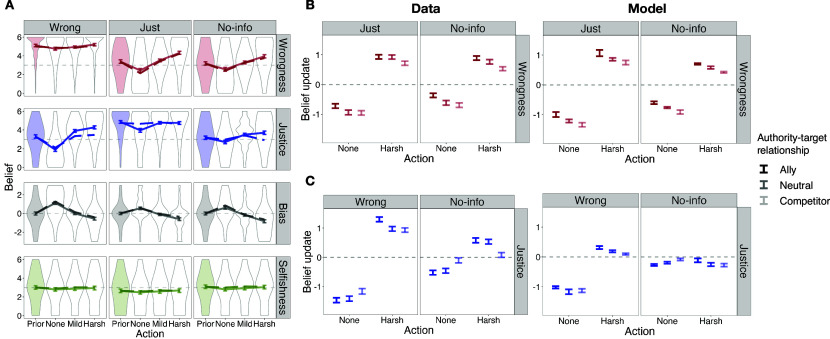
Study 2 results, across all 6 scenarios. (*A*) replication of study 1 and inverse planning model predictions, in the three conditions where no information was provided about the authority-target relationship (i.e., “Neutral”); (*B*) human and model belief updates (mean and SE of the mean across the 6 scenarios) about wrongness of the act as a function of the authority’s punitive choice, in each of the three allyship conditions, within conditions where prior beliefs about wrongness were a priori uncertain (i.e., “Just” and “No-info” conditions); (*C*) human and model belief updates about the authority’s justice motive, in each of the three allyship conditions, within conditions where prior beliefs about the authority’s justice motive were a priori uncertain (i.e., “Wrong” and “No-info” conditions). Number of observations used for calculating the SE of the humans’ belief updates in each prior condition: N=365, 355, and 352 for “Wrong,” “Just,” and “No-info” conditions, respectively; for the SE of the model’s belief updates: N=6 (i.e., number of scenarios).

**Fig. 6.**
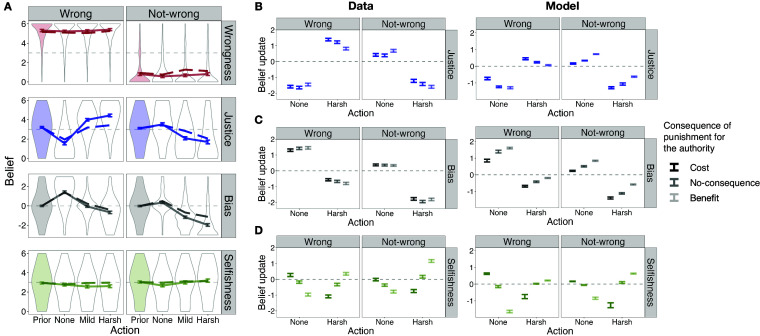
Study 3 results, across all 6 scenarios. (*A*) replication of study 1 and inverse planning model predictions, in the two conditions where punishment was described as having no direct consequences for the authority (i.e., “No-consequence” condition); (*B*–*D*) human and model belief updates about the authority’s motives as a function of the authority’s punitive choice, in each of the three consequence conditions according to experimenter labels. In all conditions, prior beliefs about the authority’s motives were uncertain. The number of observations used for finding the mean and SE of the humans’ belief updates in each prior condition is N=361. The number of observations used for finding the mean and SE of the model’s belief updates are N=6 (i.e., number of scenarios).

The key findings from study 1 were independently replicated: Observers used punishment to learn about the wrongness of the target act, depending on beliefs about the authority’s justice motive, and/or to learn about the authority’s motives, depending on the proportionality of the response to the wrongness of the target act, both in conditions that were direct replications of study 1 (Figs. 5*A* and 6*A*), and the novel conditions (Figs. 5 *B* and *C* and 6 *B* and *D*, see *SI Appendix*).

The unique conditions in studies 2 and 3 test the effects of varying priors about bias and the self-directed consequences of punishment beyond these effects. Moreover, manipulating the self-directed consequences allowed us to investigate inferences about selfishness. Indeed, both inferences about wrongness and the authority’s motives were systematically modulated by beliefs about these other variables, mostly as predicted by the inverse planning model.

### Inferences About Wrongness.

In study 2, in conditions where prior beliefs about wrongness were uncertain (“Just” and “No-info”), prior beliefs about the authority’s bias influenced inferences about wrongness (Fig. 5*B*). Observers learned more about wrongness from punishment by an ally, or doing nothing by a competitor, the responses that are unexpected given the authority-target relationship (interaction between punitive response and its unexpectedness; marginally in “Just” condition: β=0.176, std = 0.073, t(5.5) = 2.40, P=0.057; significantly in “No-info” condition: β=0.269, std = 0.075, t(13.2) = 3.61, P=0.0031).

In study 3, participants had strong prior beliefs about wrongness of the target act, and punishment only slightly changed their beliefs (punish vs. not-punish: β=0.169, std = 0.038, t(478) = 4.41, P= 1.29e-05). Varying the consequences for the authority did not modulate inferences about wrongness (interaction between response and “against-self-interest:” β=−0.007, std = 0.056, t(603) = −0.13, P=0.89).

The inverse planning model captured the nuanced dependency of belief updates about wrongness on priors about the authority’s bias (Fig. 5*B*). Overall, across all the prior conditions of studies 2 and 3, the inverse planning model (average r = 0.87) outperformed all the control models (average r = 0.70, 0.69 for the model with $U_{\text{target}}$ and the model with both $U_{\text{target}}$ and $U_{\text{self}}$ as regressors, respectively; ps < 0.001; for control models with prior beliefs as additional regressors, see *SI Appendix*, Figs. S5.1 and S5.2).

### Inferences About the Authority’s Justice Motive.

In study 2, in conditions where prior beliefs about justice motive were uncertain (“Wrong” and “No-info”), prior beliefs about the authority’s bias influenced inferences about the authority’s justice motives (Fig. 5*C*). Observers learned more about the justice motive of an ally vs. a competitor (interaction between punitive response and the authority-target relationship; “Wrong” condition: β=0.233, std = 0.073, t(4.5) = 3.21, P=0.0278 (*NP); “No-info” condition: β=0.327, std = 0.075, t(5.5) = 4.39, P=0.0058).

In study 3, prior beliefs about the authority were uncertain in all conditions, and the consequences for the authority influenced inferences about the authority’s justice motive (Fig. 6*B*). The more the punitive choice was against the authority’s self-interest, the more the authority was judged to be motivated by justice [main effect of “against-self-interest:” β=0.136, std = 0.025, t(298.8) = 5.51, P= 7.81e-08, (*NP)]. Participants’ own judgments of self-directed consequences for the authority suggested that they sometimes rejected our manipulations (*SI Appendix*, Fig. S4.4). Using participants’ own judgments in place of experimenter labels, we found that beyond this main effect (β=0.054, std = 0.014, t(13) = 3.92, P= 1.75e-03), observers more strongly updated their beliefs about the authority’s justice motive as a function of proportionality of the response, the more the punitive choice was against the authority’s self-interest and thereby unexpected (*SI Appendix*, Fig. S4.6*A*; interaction of “against-self-interest” and “unjust:” β=−0.0097, std = 0.0044, t(154.1) = −2.18, P=0.0305; not significant with experimenter labels: β=−0.003, std = 0.025, t(182.2) = −0.14, P=0.89).

The inverse planning model captured the nuanced dependency of belief updates about justice on priors about the authority’s bias (Fig. 5*C*), as well as the strengthening of justice inferences when the authority’s response is unexpected (i.e., against the authority’s self-interest; Fig. 6*B*). However, the model did not capture the main effect of “against-self-interest.” Overall, across all the prior conditions of studies 2 and 3, the inverse planning model (average r = 0.77) outperformed all the control models (average r = 0.45, 0.05; ps < 0.001).

### Inferences About the Authority’s Bias.

In study 3, the consequences for the authority modulated the effect of response and its proportionality (interaction of response and “unjust:” β=−2.156, std = 0.071, t(3959.4) = −30.36, P< 2e-16) on inferences about the authority’s bias. Observers more strongly updated their beliefs about the authority’s bias the more the punitive choice was against the authority’s self-interest and thereby unexpected, when using participants’ own judgments (*SI Appendix*, Fig. S4.6*B*; interaction of “against-self-interest,” punitive response, and “unjust:” β=−0.0169, std = 0.007, t(3502) = −2.45, P=0.0145), although this effect did not reach significance when using experimenter labels (Fig. 6*C*; β=−0.137, std = 0.087, t(3959) = −1.57, P=0.116).

The inverse planning model captured the effect of response and its proportionality as well as the strengthening of bias inferences when the response is against the authority’s self-interest (Fig. 6*C*). Overall, across all the prior conditions of studies 2 and 3, the inverse planning model (average r = 0.90) outperformed all the control models (average r = 0.81, 0.67; ps < 0.001).

### Inferences About the Authority’s Selfishness.

Study 3 also allowed us to characterize inferences about the authority’s selfishness (Fig. 6*D*). In line with prior research, the more the punitive choice (relative to the unchosen alternative) was against the authority’s self-interest, the less selfish the authority appeared (main effect of “against-self-interest:” β=−0.575, std = 0.063, t(6.3) = −9.14, P= 7.55e-05). However, attributions of selfishness were also modulated by the perceived proportionality of the punitive response. First, the more the punitive choice was unjust, the more the authority was judged to be selfish [main effect of “unjust:” β=0.295, std = 0.081, t(5.1) = 3.65, P=0.014, (*NP)]. Second, an unjust punitive choice interacted with self-interest, to strengthen inferences of selfishness (interaction of “unjust” and “against-self-interest:” β=−0.281, std = 0.078, t(5.4) = −3.59, P=0.014). All results held when using participants’ own judgments (*SI Appendix*, Fig. S4.6*C*).

The inverse planning model captured the effect of self-interest as well as the strengthening of selfishness inferences when the authority’s response is unjust and therefore unexpected (Fig. 6*D*). However, the model did not capture the main effect of “unjust” (*Discussion*). Overall, across all the prior conditions of studies 2 and 3, the inverse planning model (average r = 0.65) outperformed all the control models (average r = 0.21, 0.49; ps < 0.001).

## Shared Observations of Punishment Can Curtail, Sustain, or Spread Polarization in a Society (Study 4)

Our experiments revealed and our model explained the cognitive logic of how rational observers may interpret the same punitive choice differently, depending on their priors. We can extend the model and experiments to shed light on polarization in society.

**Fig. 7.**
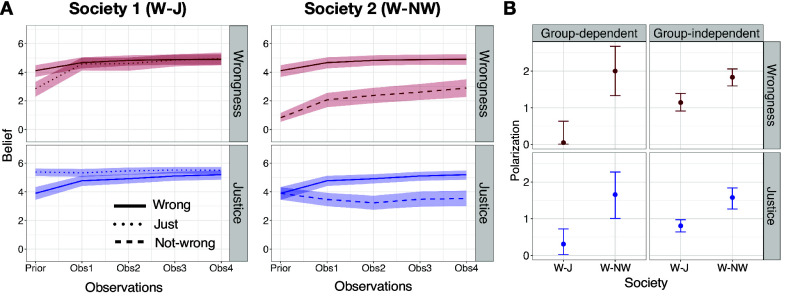
Study 4 results, belief polarization in experimental societies after shared observations of punishment. Society 1 consists of individuals in the “Wrong” and “Just” conditions, and society 2 of “Wrong” and “Not-wrong” conditions. (*A*) evolution of beliefs for the two groups within each society. The shaded ribbon shows the bootstrapped 95% CI of the mean, where “I don’t know” responses are replaced by a uniform distribution over the scale; (*B*) belief polarization within each society after four observations of harsh punishment. The group-dependent measure is the difference between the two group means (zero value indicates no difference between the group means), and the group-independent measure is the average absolute deviation from the mean (zero value indicates delta distribution). Error bars show 95% bootstrapped CI.

People in a society may have different beliefs, and authorities typically hope that repeatedly punishing norm violations will spread shared beliefs about social norms. In an experiment (Fig. 7), and our model (*SI Appendix*, Fig. S6.1), we simulated how this can happen in a society initially composed of two groups with different prior beliefs. In Society 1 (“W-J”), people in one group (“W,” N=57) had prior beliefs that a target act (e.g. “daxing”) is harmful and wrong; people in the other group (“J,” N=52) were uncertain about “daxing” but believed the authority is motivated by justice. Each participant then saw the same vignette: On four consecutive days, presented separately as four trials, the authority harshly punished four people who “daxed” (e.g., “On Monday, Paji found Tudo daxing. Paji decided to take away all of Tudo’s jats;” see *SI Appendix*). We used our cognitive model to simulate how people’s beliefs about the act’s wrongness and the authority’s justice motive would evolve, assuming all observers rationally update their beliefs after each observation and the posterior beliefs become the prior beliefs for the next punitive choice (see “Polarization simulations” in *SI Appendix*). In Society 1, the model predicted and we observed belief convergence. By the end of the vignette, people in both groups believed that daxing was wrong and the authority was just (Fig. 7*A*; main effect of condition after four observations on wrongness: β=0.220, std = 0.282, t = 0.78, P=0.437; justice: = 0.276, std = 0.190, t = 1.45, P=0.149). Polarization of beliefs about wrongness and the authority in this society decreased over time (*SI Appendix*, Fig. S6.2*B*) and was low after four punishments, measured by the distance between the average belief across groups (i.e., group-dependent; wrongness: mean = 0.046, 95% CI = [0.014, 0.637]; justice: mean = 0.307, 95% CI = [0.011, 0.686]) or by statistical dispersion of beliefs in the whole society (i.e., group-independent; wrongness: mean = 1.143, 95% CI = [0.927, 1.408]; justice: mean = 0.805, 95% CI = [0.636, 0.945]).

In contrast, our model also predicts conditions under which shared observation of punishment may not resolve the divide in a society and could even spread the initial polarization to other beliefs. An example is a society where people are initially divided in their moral beliefs about an action (e.g., jaywalking, abortion, smoking in public). In Society 2 (“W-NW”), people in one group (“W,” same as above) had prior beliefs that daxing is harmful and wrong, but people in the other group (“NW,” N=56) had prior beliefs that daxing is harmless and not wrong; both groups were initially uncertain about the authority’s motives. All participants then saw the same shared evidence of the authority’s harsh punishment of daxing on four consecutive days. In this society, our model predicted (*SI Appendix*, Fig. S6.1) and our data showed that consecutive shared observations of harsh punishment by an authority were significantly less effective in creating shared beliefs about the target action (main effect of condition after 4 observations: β=−2.040, std = 0.359, t = −5.68, P= 1.16e-07). Compounding this pattern, this society ended up additionally polarized in beliefs about the authority’s motives (Fig. 7*A*; β=−1.717, std = 0.322, t = −5.33, P= 5.42e-07). Both group-dependent and group-independent measures of polarization showed that after four observations, Society 2 remained polarized in moral beliefs about the action (group-dependent: mean = 2.00, 95% CI = [1.318, 2.707]; group-independent: mean = 1.832, 95% CI = [1.565, 2.073]), and became additionally polarized in beliefs about the authority (group-dependent: mean = 1.657, 95% CI = [1.042, 2.314]; group-independent: mean = 1.578, 95% CI = [1.264, 1.836]; Fig. 7*B*, and see *SI Appendix* for all the preregistered statistical analyses).

In these experiments, we induced differing prior beliefs in simulated societies. At the same time, participants in our studies have preexisting variation in their attitudes toward authorities. For example the Right Wing Authoritarianism (RWA) scale measures individuals’ desire for group conformity, deference to authority figures and desire to punish those who violate social norms (72). We used data from Study 1 to test the relation between RWA and participants’ prior beliefs, and then predict the resulting risks of belief polarization among people with high vs. low RWA. Before observing any punitive response, overall, people higher in RWA judged the authority in our hypothetical vignettes as more motivated by justice (β=0.133, std = 0.049, t(375.5) = 2.73, P=0.0066) and the target act as more wrong (β=0.233, std = 0.046, t(444) = 5.01, P= 7.87e-07; for results in individual conditions, see *SI Appendix*, *Results*). We then asked what happened when the authority harshly punished an act that was described as somewhat wrong (that is, a situation open to multiple interpretations): the correlation between RWA and these beliefs persisted after observing punishment (*SI Appendix*, Fig. S7.1 *B* and *C*). Using our cognitive model, we simulated how beliefs would evolve, if the authority punished the target act four more times. In these simulations, belief polarization either persisted or was amplified with more shared observations (*SI Appendix*, Fig. S7.1*D*).

In summary, any punishment can be interpreted in multiple ways, so observing the same punishment can confirm divergent prior beliefs. Our model predicts the circumstances under which more shared observations of the authority’s punitive choices can sustain polarization between individuals with different moral beliefs or political preferences and attitudes toward authorities, even when everyone is rationally updating their beliefs.

## Discussion

People learn from punishment, but what they learn varies. Many of the empirical results reported here have previously been observed separately and modeled separately. By observing them together, we uncovered the interdependence of each inference on the others; and by modeling them together, we synthesized these disparate patterns in a single framework. Rational Bayesian inference in a single cognitive model parsimoniously produced the same range of contrasting interpretations of punishment as people did, generating striking qualitative and quantitative fits to human data across a wide range of settings and measures.

First, punishment in our experiment sometimes communicated that, and how much, an act violates social norms (6, 17, 53). Our model and data illuminate the conditions for this communication to occur: when observers are confident that the authority is motivated by justice (46, 73), and uncertain about the norms. An authority who is seen as allied with or favoring the target can more effectively communicate norms by punishing than can a competitor (74–76).

Second, punishment in our model and data sometimes enhanced the authority’s reputation (36, 41). An authority who punishes proportionately to the perceived wrongness of the target act is perceived as more impartial, and more motivated by justice. These inferences correspond to the intuitive conception of a legitimate authority as impartial and benevolent (8). However, at the same time, punishment in our experiment sometimes damaged the authority’s reputation, also replicating prior evidence (33, 38). Both punishing too harshly and failing to punish harshly enough (77, 78) made authorities appear less concerned with justice, and more motivated by bias albeit biased in opposite directions.

Third, costly punishment in our experiment implied the authority was unselfish (e.g., refs. 29 and 36), whereas self-benefiting punishment implied the authority was selfish (39, 51, 78). Nevertheless, our experiments revealed a more nuanced interdependence between self-directed consequences of punishment and its severity, not previously documented. Perceptions of the authority’s selfishness were additionally influenced by the proportionality of the punitive response (79). Our current inverse planning model partially captured and explained the logic of this interdependence through the trade-off between motives in the intuitive theory of the authority. However, there remained a mismatch between the model and human judgments which could be addressed in future models by considering a more direct interdependence between people’s concepts of “selfishness” and “justice.”

Our key contribution is to synthesize these disparate observations and inferences in a single experimental framework and rational inference model and reveal how each depends critically on the other. Rather than investigating ultimate mechanisms for the evolutionary stability of punishment (e.g., ref. 25), our model characterizes the proximate computations that happen in the observer’s mind when interpreting a punitive event. To do so, we adapt a standard model of understanding others’ actions (54, 56). Inverse planning over a structured intuitive theory of the authority’s decision-making allowed us to quantitatively capture the whole range of joint belief updates. Reasoning about the utility of proportionality (i.e. the authority’s motive for justice) served as the fulcrum of joint inferences of wrongness and the authority’s motivations.

Our cognitive model systematically outperformed alternative models based on prior literature. The alternative models captured useful regularities: E.g., punishment makes the punisher appear unselfish (36, 52), or harsh punishment signals the norm violation of the target act (53). Yet these alternative models did not capture the interdependence of belief updates or generalize across contexts.

Joint inference in our cognitive model also predicts how, and why, group differences in beliefs can become and stay polarized, even while people observe the same evidence and update their beliefs rationally (80). These results reveal why punishment often fails to create shared norms and reduce societal polarization, contrary to authorities’ aspirations and best intentions (44). Here, observed punishment never fully backfired by increasing beliefs that the punished act was morally right. To predict backfiring, the model and experiment would need to include observers who have a prior belief that an authority could gain utility specifically by repressing morally right actions (81).

Additionally, there remain key limitations both of the experiment and of the model. First, there are limitations of the experimental design. In order to exert experimental control over observers’ priors, we presented hypothetical vignettes set in imaginary societies, a methodology that has been used extensively to establish cognitive mechanisms in related domains (e.g., refs. 82–84). Moreover, in order to test the quantitative predictions of the model, we directly measured people’s beliefs rather than the indirect influence of their beliefs on their behavior. However, reported beliefs may not always align with behavior in social contexts (85, 86). Future studies could test the robustness of the empirical results in more realistic scenarios, and measure changes in behavior (29, 87, 88). Finally, we conducted these experiments in a limited population of native English-speaking adults in the United States. Thus it remains an open question whether the inferences characterized here would be shared by adults in other societies (19, 89–91); and whether children rely on these inference processes when learning the social norms of their society (92).

Second, there are many potential extensions of the model instantiated here. The inverse planning framework is general and flexible as a model of observers’ inferences. To experimentally test this framework, we focused on a minimal set of authority’s punitive motives and features of punishment (e.g., severity) that we hypothesized would capture key tradeoffs in observers’ inferences. Future models could build upon and extend our current inverse planning model by i) incorporating considerations of the authority’s power or efficacy to enforce the punishment (31, 93), ii) distinguishing prior beliefs about the motives of second-party (i.e., victim) vs. third-party punishers (94), and of peers vs. authorities (38, 95), iii) differentiating between punishment aimed at justice restoration, specific deterrence, or general deterrence (32, 96; see also *SI Appendix*, Fig. S2.5 for pilot results on judgments of the authority’s deterrence motives and how they differ from judgments of the authority’s justice), and iv) comparing punishment to other ways of communicating norms, like reprimands or compensation (97). Our model could also shed light on how asymmetric prior beliefs of authorities (who believe they are motivated by justice) vs. the targets of punishment (who believe their acts were not very wrong) lead to persisting differences of interpretation in many instances of punishment (98).

Importantly, our model of observers can be extended into a model of authorities who strategically anticipate the inferences of observers. Authorities deliberately select punishments to induce desired inferences and avoid undesired ones. For example, people avoid inflicting deserved proportional punishment if observers are likely to infer the punisher was acting selfishly (39), and people tailor their punitive choices to the preferences of the audience (34). Strategic authorities may also select punishments in order to increase observers’ perception of their impartiality or justice motive (74, 99), as a means of legitimation (8). A cognitive model of strategic legitimation could embed the current model of observer inferences recursively inside a model of authorities’ planning (100, 101).

Characterizing how observers reason about and interpret the same punitive choice, in light of their different priors, has implications outside the lab. The inferences captured by our model could serve as a mechanism for norm internalization and consequently voluntary compliance with legitimate authorities (57, 93, 102, 103). When people believe an act is wrong, they avoid engaging in the act even when the probability of punishment is low. If legitimate punishment successfully communicates which acts are wrong, and punishing wrong acts sustains legitimacy, then the frequency of violations and punishments could both decline in a virtuous cycle of norm compliance and legitimation. Yet our model predicts, and our experiments show, that naturally occurring mismatches in people’s priors can lead to rationally contrasting interpretations of the same punitive choice, preventing this virtuous cycle (48, 104).

The phenomena in our experiments and model are familiar. If a judge sentences an accused man to a long prison sentence, observers with different prior beliefs may make systematically different inferences (2): Observers with prior beliefs in the impartiality and justice motives of the judge and the criminal justice system may infer that the accused man committed a serious transgression, while observers with prior beliefs in the (e.g. racial) bias of the judge or the relative harmlessness of the man’s actions may infer that the judge is not motivated by justice. Failures to punish are similarly ambiguous. If a university leader chooses not to suspend students for occupying a building in a political protest, observers with prior beliefs in the impartiality and justice motives of the university leaders may infer that the students’ protest was not very harmful, while observers with prior beliefs in the danger and harm of the political protest instead lose faith in the impartiality of the university leaders. In real-life settings, measuring potential systematic differences in priors (for example across people with different political attitudes) would allow the current model to predict the variation in what people learn from punishment.

In sum, in a single paradigm, we measured empirically and then explained formally several key inferences that people make from observed punishment. Characterizing how people jointly infer wrongness and motives can illuminate why real-world punishment attempts may fail or even backfire, contrary to authorities’ aspirations and best intentions.

## Materials and Methods

All studies used the same experimental paradigm, basic vignettes, data collection procedure, and overall analysis plan. Predictions, designs, and analysis plans for studies 1 to 3 were preregistered simultaneously at: https://osf.io/hjcrz, and study 4 at: https://osf.io/kngu9, all prior to data collection. Study procedures were approved by the MIT Committee on the Use of Humans as Experimental Subjects and we obtained informed consent from all the participants. All the data and code for the analyses and computational modeling are available at: https://osf.io/6x2we/ (107). For more detailed explanations of the methods, see *SI Appendix*.

### Participants.

The participants in all four studies were English-speaking adults in the United States recruited via online research participation platform Prolific.com. The studies and their corresponding manipulation checks (*SI Appendix*) were run serially, and participants who took part in earlier studies were not allowed to participate in the later studies. For demographic information, see *SI Appendix*.

### Dependent Variables.

In studies 1 to 3, for each of the three possible punitive responses of the authority (harsh, mild, none), after measuring the participants’ posterior beliefs (about justice, bias, selfishness, wrongness in the same order for all scenarios and participants), we also measured participants’ estimates of the harshness of the response (“[Authority] [Response] is … −3: too lenient, 0: proportional/fair, 3: too harsh”), its consequences for the target (“How much will [Target] be hurt because of [Authority] [Response]? 0: not at all, 6: very much”) and the authority (“If [Authority] decides to [Response], what do you think are the direct consequences for [Authority]? −3: huge costs, 0: no consequences, 3: huge benefits”).

### Data Analysis and Auxiliary Variables.

Across studies 1 to 3, we modeled belief updates (posterior - prior) as the primary outcome using mixed-effects linear or logistic regressions (lme4/lmerTest) with maximal random-effects structures (pruned only if needed for convergence; 105). “I don’t know” responses were scored at each scale’s midpoint, and in some analyses, mild and harsh punishments were collapsed into “punish.” In study 4, we used simple linear regressions with the same midpoint substitution.

In study 1, experimenter-defined prior conditions and punitive actions were dummy-coded. In studies 2 and 3, punitive action was coded numerically as “None” = −0.5 and “Punish” = 0.5; allyship condition as “Competitor” = −1, “Neutral” = 0, “Ally” = 1; Uself.condition as “Cost” = −1, “No-consequence” = 0, “Benefit” = 1; “Not-wrong” = −1, “Wrong” = 1. We constructed three auxiliary variables, which either used coded variables or participants’ own judgments:

[1] $$\begin{array}{cc} relationship.violation= & I_{\left[action=none\right]}\times -bias.prior\\ & +I_{\left[action=punish\right]}\times bias.prior \end{array}$$

[2] $$\begin{array}{cc} unjust=I_{\left[action=none\right]} & \times wrongness.prior\\ & +I_{\left[action=punish\right]}\times -wrongness.prior \end{array}$$

[3] $$\begin{array}{cc} against.self.interest= & I_{\left[action=none\right]}\times \Delta U_{\text{self}}\\ & +I_{\left[action=punish\right]}\times -\Delta U_{\text{self}}, \end{array}$$

where $\Delta U_{\text{self}}$ is the difference between the $U_{\text{self}}$ of punishing and doing nothing.

### Model Formalization.

To formalize observers’ intuitive theory of how authorities make punitive decisions, we wrote the authority’s expected utility over each punitive response “p” as

[4] $$\begin{array}{cc} U_{\text{total}}\left(p\right)= & \alpha_{\text{target}}U_{\text{target}}\left(p\right)+\\ & \alpha_{0}\alpha_{\text{justice}}U_{\text{justice}}\left(p\right)+\alpha_{1}\alpha_{\text{self}}U_{\text{self}}\left(p\right), \end{array}$$

where $U_{\text{target}}$ is the utility associated with the consequences of “p” for the target. Punishing is harmful, so $U_{\text{target}}$ is negative, more so for more severe punishments, and zero for doing nothing. $U_{\text{self}}$ is the utility associated with self-directed consequences of “p” for the authority (positive when “p” benefits the authority and negative when it is costly).

$U_{\text{justice}}$ denotes the extent to which “p” restores justice and is defined as the balance between the consequences of “p” for the target and wrongness of the target act; i.e., $U_{\text{justice}}$ is highest when the response is proportional to the wrongness of the act, and decreases the more the response is either too lenient or too harsh.

[5] $$\begin{array}{c} U_{\text{justice}}\left(p\right)=\left\{\begin{array}{cc} harshness(p) & harshness(p)\leq 0\\ -\gamma \;harshness(p) & 0\leq harshness(p) \end{array}\right. \end{array}$$

$$harshness\left(p\right)=\eta_{w}\;wrongness-\eta_{t}\;U_{\text{target}}+h_{0}$$

$\alpha_{0}$, $\alpha_{1}$, γ, $\eta_{w}$, $\eta_{t}$ and $h_{0}$ are constant parameters.

Authorities with different motives differ in the weight they place on each of these utilities. An authority’s justice motive ($\alpha_{\text{justice}}$) and selfishness ($\alpha_{\text{self}}$) are sampled continuously between 0 and 1, and bias toward the target ($\alpha_{\text{target}}$) is sampled between −0.5 and 0.5 to denote bias against and in favor of the target, respectively.

An authority plans whether and how severely to punish by comparing the overall utilities of possible response options using a softmax decision rule, whereby the parameter controls the noisiness, or the degree of rationality of the authority in choosing the decision with the highest utility.

[6] $$\begin{array}{cc} P(p|wrongness,\alpha_{\text{target}},\alpha_{\text{justice}}, & \alpha_{\text{self}},U_{\text{target}},U_{\text{self}})\propto \\ & \exp(\beta U_{\text{total}}\left(p\right)) \end{array}$$

By inverting this generative model of an authority’s punitive decision-making, observers simultaneously update their beliefs about the wrongness of the target act and the authority’s motives. That is, an observer uses Bayesian inference to update their beliefs about both the target act and the authority, given their appraisals of how harmful each response is to the target (i.e., $U_{\text{target}}$) and the direct consequences of each response for the authority (i.e., $U_{\text{self}}$), critically, in light of their prior beliefs about the wrongness of the act and the authority’s motives.

[7] $$\begin{array}{c} P(wrongness,\alpha_{\text{target}},\alpha_{\text{justice}},\alpha_{\text{self}}|p,U_{\text{target}},U_{\text{self}})\propto \\ P(p|wrongness,\alpha_{\text{target}},\alpha_{\text{justice}},\alpha_{\text{self}},U_{\text{target}},U_{\text{self}})\\ P(wrongness,\alpha_{\text{target}},\alpha_{\text{justice}},\alpha_{\text{self}}), \end{array}$$

where $P(p|wrongness,\alpha_{\text{target}},\alpha_{\text{justice}},\alpha_{\text{self}},U_{\text{target}},U_{\text{self}})$ is the authority’s policy derived above.

This equation highlights how observers’ belief updates depend on their prior beliefs about both the wrongness of the act and the authority’s motives, as well as the punitive context (e.g., the characteristics of the authority’s punitive response). Note that in these equations, “p” can be any punitive response, e.g., doing nothing or punishing with different severities.

### Model Implementation.

We implemented our inverse-planning model in WebPPL (v 8.9.1) and used Python (3.7.6) and R (4.1.1) for processing simulations and plotting. Observers’ beliefs about wrongness and an authority’s motives were each represented as beta distributions (shifted by −0.5 for bias), parameterized by mean (belief) and SD (uncertainty). Other classes of belief distribution can be modeled without changing any other computational machinery.

### Model Fitting and Generalization.

The joint inference model takes as inputs the utilities of each punitive action ($U_{\text{target}}$, $U_{\text{self}}$), prior-belief distributions over wrongness and motives, and a set of constant parameters ($\alpha_{0}$, $\alpha_{1}$, γ, $\eta_{w}$ and $\eta_{t}$, $h_{0}$). In estimating the model inputs and finding the best-fitting parameters, our philosophy was to push the model toward generalizability across scenarios, studies, and prior conditions, as much as possible.

We estimated $U_{\text{target}}$ by pooling judgments across all scenarios and prior conditions, and $U_{\text{self}}$ separately within each cost/benefit condition (pooling “no-consequence” conditions across studies 1 to 3, and “Cost”/“Benefit” only from study 3).

In defining the justice term, we assumed a general concept of justice that applies to all scenarios and contexts in all the studies. To capture how harm and wrongness balance against each other in forming perceived harshness, we fit a mixed-effects linear model (random intercepts for participant and scenario) to study 1 data in prior conditions where beliefs about wrongness were certain and extracted slopes $\eta_{w}$ and $\eta_{t}$ (with wrongness, $U_{\text{target}}$, and harshness judgments normalized to [0,1], [−1,0], and [−1,1], respectively). The remaining parameters γ and _0_ were optimized via cross-validation within study 1.

Prior distributions were estimated by fitting beta distributions to pooled participant judgments per scenario and prior condition (with “I don’t know” responses replaced by a uniform distribution). Each beta distribution was characterized by two parameters, “a” and “b.” In addition, we allowed both “a” and “b” to have a multiplicative constant that is later fit using cross-validation. This constant forced the mean of the prior belief distribution to be equal to its empirical mean, however, it allowed the variance of the distribution (i.e., the uncertainty of prior beliefs) to be fit (*SI Appendix*).

In study 1, to assess generalization, we held out each of six scenarios in turn, fitting parameters on the other five via grid search and by minimizing the combined mean-squared error across the four outcomes (wrongness, justice, bias, selfishness). The best-fitting parameters were then used to make predictions for each of the prior conditions and punitive responses within the held-out scenario. Model performance was quantified as the average cross-validated Pearson correlation (r) between those predictions and the actual data. Because the best-fitting parameters were nearly identical across all six training folds (*SI Appendix*, Table S2.1), we adopted a single, common parameter set. We then applied these fixed parameters to generate out-of-sample predictions for every prior condition and punitive response in studies 2 and 3.

To benchmark our model, we created four sets of control models—each set containing four separate linear regressions (for wrongness, justice, bias, and selfishness belief updates) with progressively richer predictors. We assess the performance of each set in study 1, using the same leave-one-scenario-out cross-validation. We then trained each set of control models on all six scenarios of study 1 to make out-of-sample predictions for studies 2 and 3, reporting average Pearson r as the performance metric.

### Polarization Measures.

We used two common measures of polarization (106): 1) a group-dependent measure that quantifies the distance between the two groups (i.e., mean belief) as defined by the experimental conditions and 2) a group-independent measure (i.e., average absolute deviation from the mean) that quantifies the level of statistical dispersion at the level of whole population without being tied to a notion of groups or subpopulations. We calculated the two polarization measures within each society prior to any and after each punishment observation. The “I don’t know. All values are equally likely” responses were replaced by a uniform distribution over the scale for calculating the polarization measures. We used bootstrapping to find the CI for these measures.

## Supplementary Material

Appendix 01 (PDF)

## Data Availability

All data and code for analyses and computational modeling have been deposited in OSF (DOI: 10.17605/OSF.IO/6X2WE) (107).

## References

[1] J. E. Cobbina-Dungy, D. Jones-Brown, Too much policing: Why calls are made to defund the police. Punishment Soc. **25**, 3–20 (2023).

[2] H. Jefferson, F. G. Neuner, J. Pasek, Seeing blue in black and white: Race and perceptions of officer-involved shootings. Perspect. Polit. **19**, 1165–1183 (2021).

[3] R. Benabou, J. Tirole, “Laws and norms” (Tech. Rep. w17579, National Bureau of Economic Research, 2011).

[4] J. Feinberg, “The expressive function of punishment” in *Shame Punishment* (Routledge, 2019), pp. 3–26.

[5] I. Primoratz, Punishment as language. Philosophy **64**, 187–205 (1989).

[6] A. Sarin, M. K. Ho, J. W. Martin, F. A. Cushman, Punishment is organized around principles of communicative inference. Cognition **208**, 104544 (2021). 33383397 10.1016/j.cognition.2020.104544

[7] C. R. Sunstein, On the expressive function of law. Univ. Pa. Law Rev. **144**, 2021–2053 (1996).

[8] T. R. Tyler, Psychological perspectives on legitimacy and legitimation. Annu. Rev. Psychol. **57**, 375–400 (2006). 16318600 10.1146/annurev.psych.57.102904.190038

[9] M. J. Crockett, Y. Özdemir, E. Fehr, The value of vengeance and the demand for deterrence. J. Exp. Psychol. Gen. **143**, 2279 (2014). 25285429 10.1037/xge0000018 PMC4242077

[10] F. Cushman, Punishment in humans: From intuitions to institutions. Philos. Compass **10**, 117–133 (2015).

[11] F. Cushman, A. Sarin, M. Ho, “Punishment as communication” in *The Oxford Handbook of Moral Psychology*, M. Vargas, J. Doris, Eds. (The Oxford Handbook of Moral Psychology, Oxford University Press, 2019), https://psyarxiv.com/wf3tz.

[12] F. Funk, V. McGeer, M. Gollwitzer, Get the message: Punishment is satisfying if the transgressor responds to its communicative intent. Pers. Soc. Psychol. Bull. **40**, 986–997 (2014). 24789809 10.1177/0146167214533130

[13] M. K. Ho, F. Cushman, M. L. Littman, J. L. Austerweil, People teach with rewards and punishments as communication, not reinforcements. J. Exp. Psychol. Gen. **148**, 520 (2019). 30802127 10.1037/xge0000569

[14] T. Lane, D. Nosenzo, S. Sonderegger, Law and norms: Empirical evidence. Am. Econ. Rev. **113**, 1255–1293 (2023).

[15] J. Marshall, D. A. Yudkin, M. J. Crockett, Children punish third parties to satisfy both consequentialist and retributive motives. Nat. Hum. Behav. **5**, 361–368 (2021). 33230281 10.1038/s41562-020-00975-9

[16] A. Molnar, S. J. Chaudhry, G. Loewenstein, “It’s not about the money. It’s about sending a message!’’ avengers want offenders to understand the reason for revenge Organ. Behav. Hum. Decis. Process. **174**, 104207 (2023).

[17] D. Nosenzo, E. Xiao, N. Xue, “The motive matters: Experimental evidence on the expressive function of punishment” (Tech. Rep. 2024–09, Monash University, Department of Economics, 2024).

[18] J. W. Buckholtz , From blame to punishment: Disrupting prefrontal cortex activity reveals norm enforcement mechanisms. Neuron **87**, 1369–1380 (2015). 26386518 10.1016/j.neuron.2015.08.023 PMC5488876

[19] K. Eriksson , Perceptions of the appropriate response to norm violation in 57 societies. Nat. Commun. **12**, 1481 (2021). 33674587 10.1038/s41467-021-21602-9 PMC7935962

[20] M. R. Ginther , Parsing the behavioral and brain mechanisms of third-party punishment. J. Neurosci. **36**, 9420–9434 (2016). 27605616 10.1523/JNEUROSCI.4499-15.2016 PMC5013189

[21] J. Heffner, O. FeldmanHall, Why we don’t always punish: Preferences for non-punitive responses to moral violations. Sci. Rep. **9**, 13219 (2019). 31519991 10.1038/s41598-019-49680-2 PMC6744396

[22] D. Sznycer, C. Patrick, The origins of criminal law. Nat. Hum. Behav. **4**, 506–516 (2020). 32094508 10.1038/s41562-020-0827-8

[23] J. Bregant, A. Shaw, K. D. Kinzler, Intuitive jurisprudence: Early reasoning about the functions of punishment. J. Empir. Leg. Stud. **13**, 693–717 (2016).

[24] L. B. Mulder, When sanctions convey moral norms. Eur. J. Law Econ. **46**, 331–342 (2018).

[25] K. Panchanathan, R. Boyd, Indirect reciprocity can stabilize cooperation without the second-order free rider problem. Nature **432**, 499–502 (2004). 15565153 10.1038/nature02978

[26] M. D. Santos, D. J. Rankin, C. Wedekind, The evolution of punishment through reputation. Proc. R. Soc. B Biol. Sci. **278**, 371–377 (2011). 10.1098/rspb.2010.1275 PMC3013410 20719773

[27] N. J. Raihani, R. Bshary, The reputation of punishers. Trends Ecol. Evol. **30**, 98–103 (2015). 25577128 10.1016/j.tree.2014.12.003

[28] I. Okada, Two ways to overcome the three social dilemmas of indirect reciprocity. Sci. Rep. **10**, 16799 (2020). 33033279 10.1038/s41598-020-73564-5 PMC7546724

[29] J. J. Jordan, D. G. Rand, Signaling when no one is watching: A reputation heuristics account of outrage and punishment in one-shot anonymous interactions. J. Pers. Soc. Psychol. **118**, 57 (2020). 30985155 10.1037/pspi0000186

[30] T. Batistoni, P. Barclay, N. J. Raihani, Third-party punishers do not compete to be chosen as partners in an experimental game. Proc. R. Soc. B **289**, 20211773 (2022). 10.1098/rspb.2021.1773 PMC8753170 35016543

[31] D. S. Gordon, S. E. G. Lea, Who punishes? The status of the punishers affects the perceived success of, and indirect benefits from, “moralistic’’ punishment Evol. Psychol. **14**, 1474704916658042 (2016).

[32] N. A. Dhaliwal, D. P. Skarlicki, J. Hoegg, M. A. Daniels, Consequentialist motives for punishment signal trustworthiness. J. Bus. Ethics **2020**, 1–16 (2020).

[33] E. W. de Kwaadsteniet, T. Kiyonari, W. E. Molenmaker, E. van Dijk, Do people prefer leaders who enforce norms? Reputational effects of reward and punishment decisions in noisy social dilemmas J. Exp. Soc. Psychol. **84**, 103800 (2019).

[34] J. J. Jordan, N. S. Kteily, How reputation does (and does not) drive people to punish without looking. Proc. Natl. Acad. Sci. U.S.A. **120**, e2302475120 (2023). 37406099 10.1073/pnas.2302475120 PMC10334795

[35] L. L. Tsai, M. Trinh, S. Liu, What makes anticorruption punishment popular? Individual-level evidence from China J. Polit. **84**, 602–606 (2022).

[36] J. J. Jordan, M. Hoffman, P. Bloom, D. G. Rand, Third-party punishment as a costly signal of trustworthiness. Nature **530**, 473–476 (2016). 26911783 10.1038/nature16981

[37] P. Wiessner, The role of third parties in norm enforcement in customary courts among the Enga of Papua New Guinea. Proc. Natl. Acad. Sci. U.S.A. **117**, 32320–32328 (2020). 33288714 10.1073/pnas.2014759117 PMC7768727

[38] K. Eriksson, P. A. Andersson, P. Strimling, When is it appropriate to reprimand a norm violation? The roles of anger, behavioral consequences, violation severity, and social distance Judgm. Decis. Mak. **12**, 396–407 (2017).

[39] T. S. Rai, Material benefits crowd out moralistic punishment. Psychol. Sci. **33**, 789–797 (2022). 35486472 10.1177/09567976211054786

[40] N. J. Raihani, R. Bshary, Punishment: One tool, many uses. Evol. Hum. Sci. **1**, e12 (2019). 37588410 10.1017/ehs.2019.12 PMC10427336

[41] D. Redhead, N. Dhaliwal, J. T. Cheng, Taking charge and stepping in: Individuals who punish are rewarded with prestige and dominance. *Soc. Pers. Psychol. Compass* **15**, e12581 (2021).

[42] P. Strimling, K. Eriksson, “Regulating the regulation: Norms about punishment” in *Reward and Punishment in Social Dilemmas*, P. A. M. Van Lange, B. Rockenbach, T. Yamagishi Eds. (Oxford University Press, 2014), pp. 52–69.

[43] B. Sun, L. Jin, G. Yue, Z. Ren, Is a punisher always trustworthy? In-group punishment reduces trust Curr. Psychol. **42**, 22965–22975 (2023).

[44] D. Balliet, P. A. M. Van Lange, Trust, punishment, and cooperation across 18 societies: A meta-analysis. Perspect. Psychol. Sci. **8**, 363–379 (2013). 26173117 10.1177/1745691613488533

[45] B. Depoorter, S. Vanneste, Norms and enforcement: The case against copyright litigation. Oreg. Law Rev. **84**, 1127 (2005).

[46] L. B. Mulder, P. Verboon, D. De Cremer, Sanctions and moral judgments: The moderating effect of sanction severity and trust in authorities. Eur. J. Soc. Psychol. **39**, 255–269 (2009).

[47] P. Verboon, M. van Dijke, When do severe sanctions enhance compliance? The role of procedural fairness J. Econ. Psychol. **32**, 120–130 (2011).

[48] J. Amemiya, E. Mortenson, M. T. Wang, Minor infractions are not minor: School infractions for minor misconduct may increase adolescents’ defiant behavior and contribute to racial disparities in school discipline. Am. Psychol. **75**, 23–36 (2020). 31081648 10.1037/amp0000475

[49] C. Bicchieri, E. Dimant, E. Xiao, Deviant or wrong? The effects of norm information on the efficacy of punishment J. Econ. Behav. Organ. **188**, 209–235 (2021).

[50] J. Del Toro, D. B. Jackson, A. Testa, M. T. Wang, The spillover effects of classmates’ police intrusion on adolescents’ school-based defiant behaviors: The mediating role of institutional trust. Am. Psychol. **78**, 941–954 (2023). 36913279 10.1037/amp0001148

[51] E. Xiao, Profit-seeking punishment corrupts norm obedience. Games Econ. Behav. **77**, 321–344 (2013).

[52] J. J. Jordan, D. G. Rand, Third-party punishment as a costly signal of high continuation probabilities in repeated games. J. Theor. Biol. **421**, 189–202 (2017). 28390842 10.1016/j.jtbi.2017.04.004

[53] K. Khalmetski, A. Ockenfels, Altruistic punishment as norm-signaling: A model and experimental evidence. SSRN, 10.2139/ssrn.4716824 (2024).

[54] C. L. Baker, R. Saxe, J. B. Tenenbaum, Action understanding as inverse planning. Cognition **113**, 329–349 (2009). 19729154 10.1016/j.cognition.2009.07.005

[55] S. D. Houlihan, M. Kleiman-Weiner, L. B. Hewitt, J. B. Tenenbaum, R. Saxe, Emotion prediction as computation over a generative theory of mind. Philos. Trans. R. Soc. A **381**, 20220047 (2023). 10.1098/rsta.2022.0047 PMC10239682 37271174

[56] J. Jara-Ettinger, H. Gweon, L. E. Schulz, J. B. Tenenbaum, The naïve utility calculus: Computational principles underlying commonsense psychology. Trends Cogn. Sci. **20**, 589–604 (2016). 27388875 10.1016/j.tics.2016.05.011

[57] T. R. Tyler, P. A. Goff, R. J. MacCoun, The impact of psychological science on policing in the United States: Procedural justice, legitimacy, and effective law enforcement. Psychol. Sci. Public Interest **16**, 75–109 (2015). 26635334 10.1177/1529100615617791

[58] R. C. Mayer, J. H. Davis, F. D. Schoorman, An integrative model of organizational trust. Acad. Manag. Rev. **20**, 709–734 (1995).

[59] R. Saxe, Perceiving and pursuing legitimate power. Trends Cogn. Sci. **26**, 1062–1063 (2022). 36150968 10.1016/j.tics.2022.08.008

[60] R. Boyd, H. Gintis, S. Bowles, P. J. Richerson, The evolution of altruistic punishment. Proc. Natl. Acad. Sci. U.S.A. **100**, 3531–3535 (2003). 12631700 10.1073/pnas.0630443100 PMC152327

[61] E. Fehr, S. Gächter, Altruistic punishment in humans. Nature **415**, 137–140 (2002). 11805825 10.1038/415137a

[62] W. Hofmann, M. J. Brandt, D. C. Wisneski, B. Rockenbach, L. J. Skitka, Moral punishment in everyday life. Pers. Soc. Psychol. Bull. **44**, 1697–1711 (2018). 29848212 10.1177/0146167218775075

[63] B. Schiller, T. Baumgartner, D. Knoch, Intergroup bias in third-party punishment stems from both ingroup favoritism and outgroup discrimination. Evol. Hum. Behav. **35**, 169–175 (2014).

[64] A. C. Weidman, W. J. Sowden, M. K. Berg, E. Kross, Punish or protect? How close relationships shape responses to moral violations Pers. Soc. Psychol. Bull. **46**, 693–708 (2020). 31535954 10.1177/0146167219873485

[65] M. Kleiman-Weiner, A. Shaw, J. Tenenbaum, “Constructing social preferences from anticipated judgments: When impartial inequity is fair and why?” in *Proceedings of the Annual Meeting of the Cognitive Science Society* (2017), vol. 39. https://faculty.washington.edu/maxkw/publication/kleiman-2017-constructing/kleiman-2017-constructing.pdf.

[66] K. M. Carlsmith, J. M. Darley, P. H. Robinson, Why do we punish? Deterrence and just deserts as motives for punishment J. Pers. Soc. Psychol. **83**, 284 (2002). 12150228 10.1037/0022-3514.83.2.284

[67] R. M. McFatter, Purposes of punishment: Effects of utilities of criminal sanctions on perceived appropriateness. J. Appl. Psychol. **67**, 255 (1982). 7107551

[68] L. Fitouchi, M. Singh, Punitive justice serves to restore reciprocal cooperation in three small-scale societies. Evol. Hum. Behav. **44**, 502–514 (2023).

[69] J. Deigh, Punishment and proportionality. Crim. Justice Ethics **33**, 185–199 (2014).

[70] U. Fischbacher, S. Gächter, S. Quercia, The behavioral validity of the strategy method in public good experiments. J. Econ. Psychol. **33**, 897–913 (2012).

[71] S. Radkani, R. Saxe, “What people learn from punishment: Joint inference of wrongness and punisher’s motivations from observation of punitive choices” in *Proceedings of the Annual Meeting of the Cognitive Science Society*, M. Goldwater, F. K. Anggoro, B. K. Hayes, D. C. Ong, Eds. (California Digital Library, University of California, Oakland, CA, 2023), vol. 45.

[72] J. Duckitt, B. Bizumic, S. W. Krauss, E. Heled, A tripartite approach to right-wing authoritarianism: The authoritarianism-conservatism-traditionalism model. Polit. Psychol. **31**, 685–715 (2010).

[73] M. de Vel-Palumbo, M. Twardawski, M. Gollwitzer, Making sense of punishment: Transgressors’ interpretation of punishment motives determines the effects of sanctions. Br. J. Soc. Psychol. **62**, 1395–1417 (2023). 36892128 10.1111/bjso.12638

[74] L. L. Tsai, When People Want Punishment: Retributive Justice and the Puzzle of Authoritarian Popularity (Cambridge University Press, 2021).

[75] A. J. Berinsky, Rumors and health care reform: Experiments in political misinformation. Br. J. Polit. Sci. **47**, 241–262 (2017).

[76] S. D. Benegal, L. A. Scruggs, Correcting misinformation about climate change: The impact of partisanship in an experimental setting. Clim. Change **148**, 61–80 (2018).

[77] J. C. Salcedo, W. Jimenez-Leal, Severity and deservedness determine signalled trustworthiness in third party punishment. Br. J. Soc. Psychol. **63**, 453–471 (2024). 37787476 10.1111/bjso.12687

[78] L. Wang, J. K. Murnighan, The dynamics of punishment and trust. J. Appl. Psychol. **102**, 1385 (2017). 28471207 10.1037/apl0000178

[79] R. W. Carlson, C. Adkins, M. J. Crockett, M. S. Clark, Psychological selfishness. Perspect. Psychol. Sci. **17**, 1359–1380 (2022). 35436157 10.1177/17456916211045692

[80] S. Radkani, M. Landau-Wells, R. Saxe, How rational inference about authority debunking can curtail, sustain or spread belief polarization. PNAS Nexus **3**, pgae393 (2024). 39411098 10.1093/pnasnexus/pgae393 PMC11475407

[81] D. A. Reinero, E. A. Harris, S. Rathje, A. Duke, J. J. Van Bavel, Partisans are more likely to entrench their beliefs in misinformation when political outgroup members fact-check claims. PsyArXiv [Preprint] (2023). 10.31234/osf.io/z4df3 (Accessed 5 September 2024).

[82] D. Nettle, R. Saxe, Preferences for redistribution are sensitive to perceived luck, social homogeneity, war and scarcity. Cognition **198**, 104234 (2020). 32062160 10.1016/j.cognition.2020.104234

[83] S. Partington, S. Nichols, T. Kushnir, Rational learners and parochial norms. Cognition **233**, 105366 (2023). 36669334 10.1016/j.cognition.2022.105366

[84] K. Skilling, G. J. Stylianides, Using vignettes in educational research: A framework for vignette construction. Int. J. Res. Method Educ. **43**, 541–556 (2020).

[85] O. FeldmanHall , What we say and what we do: The relationship between real and hypothetical moral choices. Cognition **123**, 434–441 (2012). 22405924 10.1016/j.cognition.2012.02.001 PMC3355304

[86] J. McDonald, Avoiding the hypothetical: Why “mirror experiments” are an essential part of survey research. *Int J Public Opin R*. **32**, 266–283 (2020).

[87] M. Gollwitzer, J. Braun, F. Funk, P. Süssenbach, People as intuitive retaliators: Spontaneous and deliberate reactions to observed retaliation. Soc. Psychol. Pers. Sci. **7**, 521–529 (2016).

[88] M. Gummerum, B. López-Pérez, E. Van Dijk, L. F. Van Dillen, When punishment is emotion-driven: Children’s, adolescents’, and adults’ costly punishment of unfair allocations. Soc. Dev. **29**, 126–142 (2020).

[89] K. Eriksson , Cultural universals and cultural differences in meta-norms about peer punishment. Manag. Org. Rev. **13**, 851–870 (2017).

[90] Z. H. Garfield , Norm violations and punishments across human societies. Evol. Hum. Sci. **5**, e11 (2023). 37587937 10.1017/ehs.2023.7 PMC10426015

[91] C. Molho, F. De Petrillo, Z. H. Garfield, S. Slewe, Cross-societal variation in norm enforcement systems. Philos. Trans. R. Soc. B **379**, 20230034 (2024). 10.1098/rstb.2023.0034 PMC10799737 38244602

[92] Y. E. Lee, S. Radkani, R. Saxe, Children learn what is right or wrong selectively from a legitimate authority’s punishment. OSF [Preprints] (2024). 10.31219/osf.io/56nqb_v3 (Accessed 23 April 2025). 42024340

[93] M. Levi, L. Stoker, Political trust and trustworthiness. Annu. Rev. Polit. Sci. **3**, 475–507 (2000).

[94] J. W. Martin, J. J. Jordan, D. G. Rand, F. Cushman, When do we punish people who don’t? Cognition **193**, 104040 (2019). 31408816 10.1016/j.cognition.2019.104040

[95] J. Marshall, K. Mermin-Bunnell, P. Bloom, Developing judgments about peers’ obligation to intervene. Cognition **201**, 104215 (2020). 32464406 10.1016/j.cognition.2020.104215

[96] F. Tan, E. Xiao, Third-party punishment: Retribution or deterrence? J. Econ. Psychol. **67**, 34–46 (2018).

[97] N. A. Dhaliwal, I. Patil, F. Cushman, Reputational and cooperative benefits of third-party compensation. Organ. Behav. Hum. Decis. Process. **164**, 27–51 (2021).

[98] G. S. Adams, Asymmetries between victims’ and transgressors’ perspectives following interpersonal transgressions. Soc. Pers. Psychol. Compass **10**, 722–735 (2016).

[99] A. Shaw, Beyond “to share or not to share’’ the impartiality account of fairness. Curr. Dir. Psychol. Sci. **22**, 413–417 (2013).

[100] M. K. Ho, R. Saxe, F. Cushman, Planning with theory of mind. Trends Cogn. Sci. **26**, 959–971 (2022). 36089494 10.1016/j.tics.2022.08.003

[101] S. Radkani, J. Tenenbaum, R. Saxe, “Modeling punishment as a rational communicative social action” in *Proceedings of the Annual Meeting of the Cognitive Science Society* J. Culbertson, A. Perfors, H. Rabagliati, V. Ramenzoni, Eds. (California Digital Library, University of California, Oakland, CA, 2022), vol. 44.

[102] J. Amemiya, A. Fine, M. T. Wang, Trust and discipline: Adolescents’ institutional and teacher trust predict classroom behavioral engagement following teacher discipline. Child Dev. **91**, 661–678 (2020). 30927372 10.1111/cdev.13233

[103] D. Baldassarri, G. Grossman, Centralized sanctioning and legitimate authority promote cooperation in humans. Proc. Natl. Acad. Sci. U.S.A. **108**, 11023–11027 (2011). 21690401 10.1073/pnas.1105456108 PMC3131358

[104] J. M. Gau, R. K. Brunson, Procedural injustice, lost legitimacy, and self-help: Young males’ adaptations to perceived unfairness in Urban policing tactics. J. Contemp. Crim. Justice **31**, 132–150 (2015).

[105] D. J. Barr, R. Levy, C. Scheepers, H. J. Tily, Random effects structure for confirmatory hypothesis testing: Keep it maximal. J. Mem. Lang. **68**, 255–278 (2013). 10.1016/j.jml.2012.11.001 PMC3881361 24403724

[106] A. Bramson , Understanding polarization: Meanings, measures, and model evaluation. Philos. Sci. **84**, 115–159 (2017).

[107] S. Radkani, J. Tenenbaum, R. Saxe, Joint inference of wrongness and legitimacy from observation of punitive decisions. Open Science Framework. https://osf.io/6x2we/. Deposited 23 July 2025.

