# Supplementary material for: What people learn from punishment: A cognitive model
Source: Proc Natl Acad Sci U S A. 2025 Aug 4;122(32):e2500730122. doi: 10.1073/pnas.2500730122 (PMC12358846; doi:10.1073/pnas.2500730122)
Supplement: Supplementary file 1 — Appendix 01 (PDF) [file pnas.2500730122.sapp.pdf]

Supporting Information

1. Across all studies

Scenario	Authority	Target	Target act	Means of punishment	Punitive choice
**S1**	Paji	Tudo	Daxing	People in this society have something they call 'jats', which really matter to them.	Paji could either do nothing, take away half of Tudo's jats, or take away all of Tudo's jats.
**S2**	Didi	Kiva	Zimming	People in this society sometimes get the chance to go to a place called a 'mot', which is really pleasurable for them.	Didi could either do nothing, not allow Kiva to go to the mot for a week, or not allow Kiva to go to the mot for two months.
**S3**	Fadi	Buba	Huxing	One task in this society is cleaning the deb, which is necessary but very unpleasant work.	Fadi could either do nothing, force Buba to spend ten hours cleaning the deb, or force Buba to spend one week cleaning the deb.
**S4**	Maki	Sudo	Using a voice during the day	-	Maki could either do nothing, take away the voca for the rest of the week, or take away the voca until the end of the year.
**S5**	Vivi	Lula	Timming	There is a piece of clothing in this society called xoops. A person loses access to many of the public places while wearing	Vivi could either do nothing, force Lula to wear xoops for three days, or force Lula to wear xoops for a month.
**S6**	Nadi	Zuba	Pexing	In this society, people work hard to cultivate crops and make a living.	Nadi could either do nothing, force Zuba to give away 1 barrel of food to community resources, or force Zuba to give away 2 barrels of food to community resources.

Fig. S1. Scenarios used in studies 1-3. All scenarios had the same structure as outlined in Fig. 2 in the main text. In each scenario, we used novel names for the authority, target, the target act and a novel means of punishment. Across our scenarios, punitive responses implied loss of goods, loss of privilege, imposition of an unpleasant task, etc.

Participants. Participants reported their gender (Male, Female, Other, Prefer not to answer), age (in bins of 18-24, 25-34, 35-44, 45-54, 55-64, 65 and above), race (White, Black or African-American, American Indian or Alaskan Native, Asian, Native Hawaiian or other Pacific Islander, Hispanic, Other), Right-Wing-Authoritarianism and political ideology. We measured RWA using six items (adopted from Duckitt et al., 2010) in randomized order: "Our society should have strong, tough leaders", "People who break the rules need to be treated with leniency and kindness" (reverse-coded), "People in our society need to preserve their traditional values", "Everyone should be free to pursue his or her own lifestyle" (reverse-coded), "People must be obedient to their leaders", and "People should be allowed to question and challenge decisions they do not like", each on a 9-point scale (-4: very strongly disagree to 4: very strongly agree). Each participant's RWA was found by taking the average of these 6 items (Cronbach's alpha in study 1: 0.71, study 2: 0.73, study 3: 0.75, study 4: 0.59). Self-reported ideology was measured using a 7-point likert scale (1: Extremely liberal, 4:

Moderate, 7: Extremely conservative), with two additional response options of “Don’t know” and “Haven’t thought much about it”.

In study 1, our final sample included N=358 participants (194 male, 160 female, 4 other; age range: 18 to above 65; RWA: 1st Qua.=-1.50, median=-0.667, 3rd Qua.=0.167). In study 2, the final sample included N=535 participants (278 male, 245 female, 10 other, 2 prefer not to say; age range: 18 to above 65; RWA: 1st Qua.=-1.33, median=-0.334, 3rd Qua.=0.334). In study 3, our final sample included N=361 participants (178 male, 176 female, 6 other, 1 prefer not to say; age range: 18 to above 65; RWA: 1st Qua.=-1.5, median=-0.667, 3rd Qua.=0.167). In study 4, our final sample included N=165 (80 male, 85 female; age range: 18 to above 65; RWA: 1st Qua.=-0.50, median=0.33, 3rd Qua.=0.83). On average, the participants rated themselves as slightly liberal (ideology: mean per experiment ranged from 3.19 to 4.12, and s.d.s 1.79 to 1.95; RWA: on a -4 to 4 scale, means per experiment ranged from -0.70 to 0.09, and s.d.s 1.05 to 1.33).

Manipulation check pilots. For each of the three studies, before running the main experiment, we ran a manipulation check pilot with an independent group of participants (N=30 for Study 1, N=45 for study 2, N=30 for Study 3) to confirm that the language used in our scenarios manipulated participants’ prior beliefs as intended. In the pilots, we used the exact same scenarios and conditions as in the corresponding main study. However, we adapted our dependent variables to measure both the value and the uncertainty of each individual’s priors, by asking the participants to distribute 100 votes on the relevant scale for each question. In all three studies, we confirmed that our scenarios manipulated both the value and the dispersion of participants’ prior beliefs about novel actions and authorities, as well as self-directed consequences of punishment for the authority in study 3 (Supplementary Fig. S2.3, S3.3, S3.4, S4.3).

Experiment implementation.
The survey was implemented in Qualtrics (https://www.qualtrics.com/) and Qualtrics built-in randomizer was used to randomize assignment of participants to scenario-conditions pairs as determined by a latin square design, the order of showing scenario-conditions for each participant, and the order of the three posterior measurement blocks for each scenario and participant.

Auxiliary variables and coding of discrete variables.
In study 1, experimenter-defined prior conditions and punitive actions were dummy-coded.

In study 2, we coded punitive responses as "None"=-0.5, "Punish"=0.5. In the analyses using experimenter labels for prior conditions, allyship condition was coded as "Competitor"=-1, "Neutral"=0, "Ally"=1. We defined an auxiliary variable, relationship.violation, which takes higher values when the authority's response is unexpected given their relationship with the target; that is, doing nothing especially when the authority is highly biased against the target, or punishing especially when the authority is highly biased in favor of the target. Formally, this variable was defined as

$$relationship.violation\ = I_{\{action=none\}}\ *\ R(bias.prior)\ + I_{\{action=punish\}}\ *\ bias.prior$$

where, $R(bias.prior)\ =-\ bias.prior$ and bias.prior was replaced by allyship condition (as coded above) in the analyses using experimenter labels, and replaced by the measured prior beliefs about the authority's bias in analyses using participants' own judgments. Allyship condition, relationship.violation auxiliary variable, and participants' own priors were mean-centered (without scaling) for each analysis separately.

In study 3, we coded punitive responses as "None"=-0.5, "Punish"=0.5. In the analyses using experimenter labels for prior conditions, U_{self} condition was coded as "Cost"=-1, "No-consequence"=0, "Benefit"=1, and the prior condition as "Not-wrong"=-0.5, "Wrong"=0.5. We defined two auxiliary variables: "unjust" which takes higher values when the authority's response is not proportionate to the wrongness of the target act, that is doing nothing in response to wrong acts and punishing in response to acts that are not wrong; and "against.self.interest" which takes higher values the more the authority's chosen response is costlier than the alternative response, that is doing nothing when punishment is beneficial or punishing when punishment is costly. Formally, these variables were defined as

$$unjust\ = I_{\{action=none\}}\ *\ wrongness.prior\ + I_{\{action=punish\}}\ *\ R(wrongness.prior)$$

and, $against.self.interest\ = I_{\{action=none\}}\ *\ \Delta U_{self}\ + I_{\{action=punish\}}\ *\ R(\Delta U_{self})$. In these equations, ΔU_{self} is the difference between the U_{self} of punishing and doing nothing, $R(\Delta U_{self})\ =-\ \Delta U_{self}$ and $R(wrongness.prior)\ =-\ wrongness.prior$. In the analyses using experimenter labels, ΔU_{self} and $wrongness.prior$ were replaced by experimental conditions (as coded above). In the analyses using participants' own judgments, ΔU_{self} was replaced by the difference between U_{self} judgments of punishing and doing nothing, and $wrongness.prior$ was replaced by the measured prior beliefs about the wrongness of the target act. The two auxiliary variables were mean-centered (without scaling) for each analysis separately.

Model fitting.

To make predictions about the posterior beliefs, the joint inference model takes as input the utilities associated with each punitive choice (i.e., U_{target}, U_{self}), the distribution of prior beliefs over wrongness and the authority's motives, as well as a set of constant parameters (α_0, α_1, β, γ, h_0, η_w, η_t). In estimating the model inputs and finding the best fitting parameters, our philosophy was to push the model towards generalizability across scenarios, studies and prior conditions, as much as possible.

To ensure generalization across prior conditions, we estimated the utilities associated with each novel and unique means of punishment introduced in each scenario by pooling the data from as many prior conditions across all studies as the design allowed (e.g., we averaged judgments in all the prior conditions in all three studies to find U_{target} of taking all of Tudo's jats in scenario 1). We estimated U_{self} of the punitive responses within each U_{self} condition. To estimate U_{self} of the three punitive responses in conditions where punishment was described as having no-consequences, we pooled the data from studies 1-3. To estimate U_{self} in "Cost" and "Benefit" conditions, we only used the data from study 3.

In defining the justice term, we assumed a general concept of justice that applies to all scenarios and contexts in all the studies. To formalize the concept of justice, we first estimated how U_{target} and wrongness are balanced against each other to form the harshness judgments (indicated by the η_w and η_t parameters). We fit a mixed effects linear regression model to all participants' judgments of the harshness of punishment, using their individual judgements of wrongness and harm imposed on the target as independent variables. We controlled for random intercepts of participant and scenario. We were primarily interested in the fixed slopes as they show how the harm imposed on the target by each response is balanced against the wrongness of the target act. To find η_w and η_t parameters, we only used the data from study 1, and only from the prior conditions where beliefs about wrongness were certain (i.e., "Wrong", "Somewhat-wrong" and "Not-wrong"), because 1) we varied beliefs about wrongness over a wide range of the scale, 2) the prior beliefs did not change substantially as a result of observing the punitive response, and 3) there were very few "I don't know" responses for beliefs about wrongness. Wrongness, U_{target} and harshness judgments were normalized between [0,1], [-1,0] and [-1,1], respectively. The two additional parameters of γ, h_0 were fit using cross-validation across the whole dataset of study 1 (see below).

To estimate the prior belief distributions, although we did not have access to each individual participants' belief distribution (we asked each participant to report a single value for their belief), we had the distribution of prior and posterior beliefs in our sample population for each combination of scenario, prior condition and punitive response, separately (e.g.,

distribution of posterior beliefs in study 1, scenario 1 in the “Wrong” prior condition if the authority chooses to do nothing). Therefore, we used our model to explain the inferences of the “average participant”, whose belief distribution is the distribution of that belief in our sample population.

Within each study, we first fit a beta distribution to the pool of participants’ judgments in each scenario and prior condition, separately for each dependent variable. We considered “All values are equally likely” responses as a uniform distribution over the whole scale. Each beta distribution was characterized by two parameters, ‘a’ and ‘b’. In addition, we allowed both ‘a’ and ‘b’ to have a multiplicative constant which we later fit using cross-validation (see below). This constant forced the mean of the prior belief distribution to be equal to its empirical mean, however, it allowed the variance of the distribution (i.e., the uncertainty of prior beliefs) to be fit. This parameter accounted for the fact that we only had access to the distribution of belief values across our sample, which may not reflect the true uncertainty of individuals’ beliefs, as reflected in their willingness to update their beliefs based on the evidence. To decrease the model’s degrees of freedom, we did not have a unique constant for every prior distribution, however. The prior distribution over each dependent variable was assigned a constant depending on whether there was any information provided about that variable or the prior belief was kept uncertain. For example, in study 1 and for the wrongness beliefs, “Wrong”, “Somewhat-wrong” and “Not-wrong” conditions had the same constant which differed from the shared constant of “Just”, “Unjust” and “No-info” conditions. Supplementary Fig S1.5 shows which parameter corresponds to which dependent variable and prior condition.

Finally, to ensure generalization across studies, we only fit the model parameters using the data from study 1, and fix the model parameters in generating predictions in studies 2 and 3. Given the novel prior conditions in studies 2 and 3, this procedure tested the ability of the model to generalize to never-seen-before contexts. To ensure generalization across scenarios, we assessed model performance within study 1 using a cross-validation procedure. We held one scenario out as the test data, and found the best fitting parameters on the remaining five scenarios (see “Grid search” section). The best fitting parameters were then used to find the model predictions for posterior belief values in all the prior conditions within the held-out scenario.

We fit all the parameters of the model in study 1, i.e., α_0, β, γ, h_0, η_w , η_t as well as the constants controlling the variance of prior belief distributions. The best fitting parameters were found on each training set (i.e., all the prior conditions within five scenarios) by minimizing the sum of the mean-squared-error of wrongness, justice, bias and selfishness, to

capture all 4 dependent variables simultaneously. The best fitting parameters were then used to make predictions for all the prior conditions and punitive responses within the held-out scenario. This procedure was repeated for each of the 6 scenarios (as the held-out scenario), and we report the average cross-validated r between model predictions and held-out data as the final measure of model performance. In studies 2 and 3, we used the model fit in study 1 to make predictions for all the prior conditions and punitive responses within each of the 6 scenarios. Average r was again used as the final measure of model performance.

For fitting and assessing the control models, a similar procedure was used. We had four sets of control models. Each set consisted of four linear regression models that were separately optimized to predict belief updates about wrongness, justice, bias and selfishness. The four sets of control models differed in the regressors they used. The first set used only the punitive harm imposed on the target (U_{target}) of each response within each scenario as a regressor. The second set used both the consequences of each response for the target (U_{target}) and the authority (U_{self}). The third and fourth sets additionally used the mean of prior belief distribution over each dependent variable (within each scenario and prior condition separately) to predict belief updates about that variable. In study 1, to find the average r of each control model, we used a similar cross-validation procedure as above. To assess the generalization ability of the control models, we fit the models on all the data from study 1 (all 6 scenarios). We then used the fitted models to make predictions for the unseen data in studies 2 and 3. Average r (across the 6 scenarios) was reported as the final measure of model performance.

Grid search.

In study 1, we fit α_0, β, γ, h_0 on each training set (consisting of 5 scenarios) using a coarse grid-search over [0.5, 0.75, 1], [11, 13], [0.6, 1, 1.4], [-0.175, -0.15], respectively. The choice of these values was guided by the model fits in an independent dataset (Radkani & Saxe, 2023). The multiplicative parameters controlling the variance of the beta distributions, i.e., C1 to C5, were optimized simultaneously as the other parameters, using a grid search over [0.5, 1, 2], [0.5, 1, 2, 3], [1, 3, 5], [0.5, 1, 2], and [0.5, 1, 2], respectively. The best fitting parameters were largely similar among the 6 training subsets of the data. α_1 was fixed to be 1. The best fitting parameters within each training set are reported in Supplementary table S2.1.

To make predictions in studies 2 and 3, we fixed the model parameters, using values that generated the best fit in all or almost all the training sets in study 1; these parameters were $\alpha_0 = 0.75, \alpha_1 = 1, \beta = 12, \gamma = 1.4, h_0 = -0.15, C_1 = 1, C_2 = 2, C_3 = 5, C_4 = 1,$

$C_5 = 2$. Indeed, the best fitting β was 11 in exactly half of the training sets, and 13 in the other half; so we chose 12 as the mean of the best fitting βs.

Model comparison (bootstrapping).

To statistically compare the inverse planning model versus each set of control models, we used bootstrapping. In study 1, we sampled with replacement from all the scenario, prior condition and action combinations (6 * 6 * 3 = 108 observations), for 1000 times. We then fit the inverse planning model and the control models on each sampled dataset, and found the difference between the performance (i.e., r) of the inverse planning model and each set of control models. In studies 2 and 3, we sampled with replacement from all the scenario, prior condition and action combinations, for 1000 times. We then found the difference between the performance (i.e., r) of the inverse planning model and the control models that were all fit in study 1, on each sampled dataset.

2. Study 1

Materials and Design.

Prior condition	Justice information	Wrongness information	Allyship information	Direct consequences for authority
**Not-wrong**	-	You hear that daxing is pretty common, and lots of people are frequently daxing. Daxing does not bother people.	-	Either doing nothing or taking away Tudo's jats has no direct costs or benefits for Paji.
**Somewhat-wrong**	-	You hear that people occasionally dax, but mostly avoid it. Daxing inconveniences and annoys many people.		
**Wrong**	-	You hear that daxing is very rare, almost no one ever daxes. Daxing is very harmful to other people.		
**Just**	You hear that Paji has a strong sense of justice and tries to make sure everyone is treated fairly.	You don't know anything about daxing.		
**Unjust**	You hear that Paji is not particularly concerned with justice or fairness.	You don't know anything about daxing.		
**No-info**	-	You don't know anything about daxing.		

Fig. S2.1: Wording of manipulations across the six conditions of study 1. The same wording was used for all six scenarios, with the corresponding names used for the authority, the target and the target act.

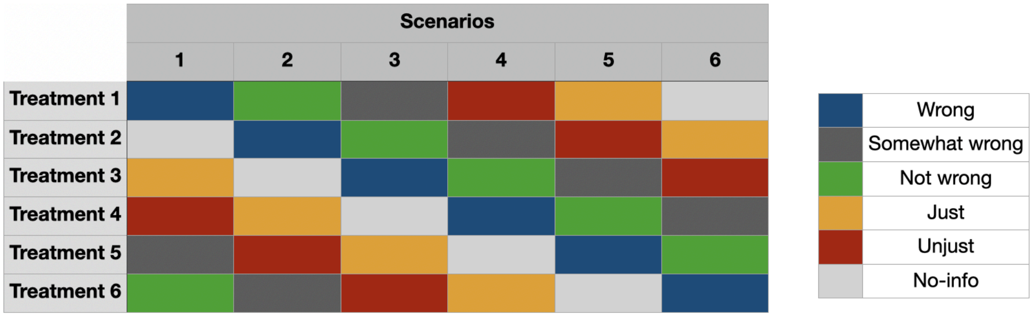

Fig. S2.2 Counterbalancing of scenarios and conditions across participants using a latin square design. Each participant was randomly assigned to a treatment (i.e., one row of this matrix) by the Qualtrics randomizer. The order of the scenarios were also randomized within each participant.

Manipulation check pilot.

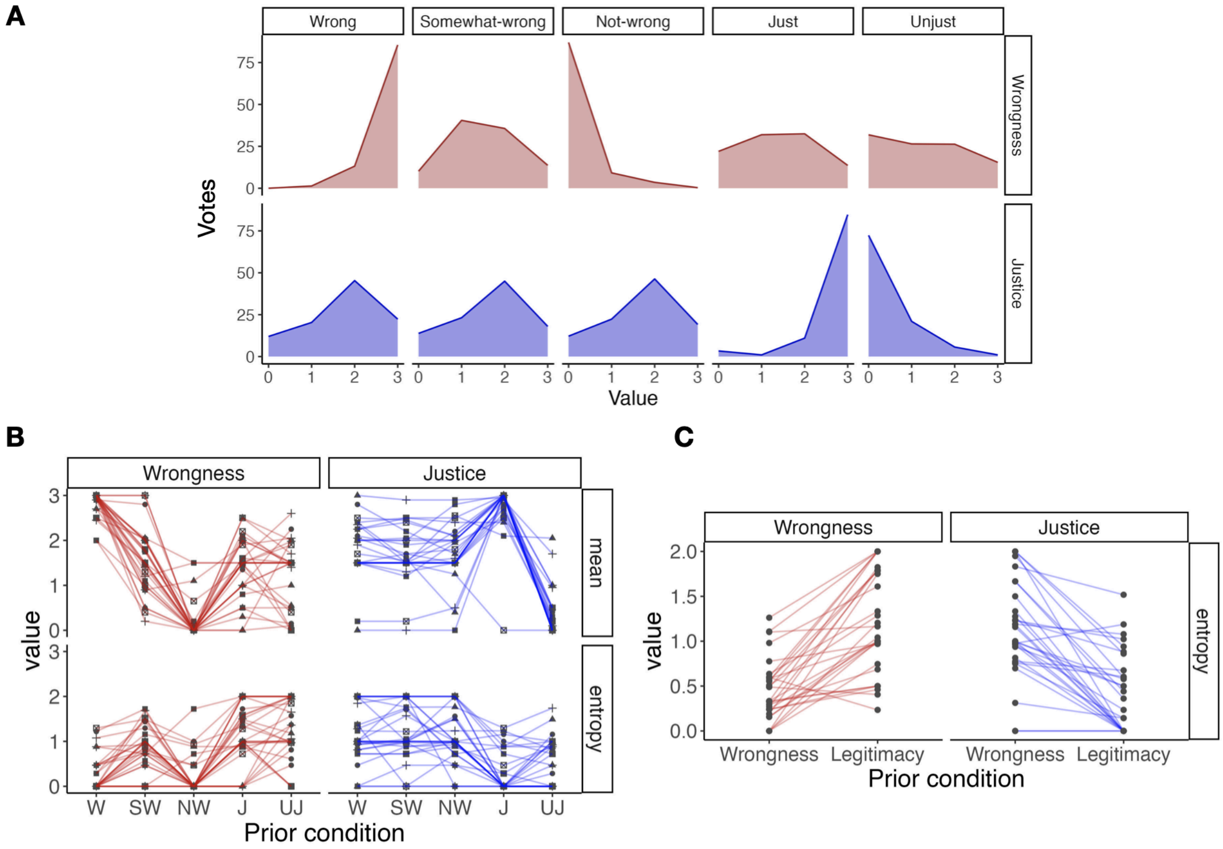

Fig. S2.3 Participants' prior beliefs in a manipulation check pilot study (N=30). The pilot used the same design as study 1, except that we measured both the value and uncertainty of participants' priors by asking the participants to distribute 100 votes on the relevant scale for each question; A) average of all participants' belief distributions; B) mean and entropy of participants' belief distributions. Each participants' belief statistics across different prior conditions is connected with a line; C) entropy of participants' belief distributions averaged across conditions where either wrongness (W, SW, NW) or the authority's legitimacy (J, UJ) were manipulated.

Supplementary results.

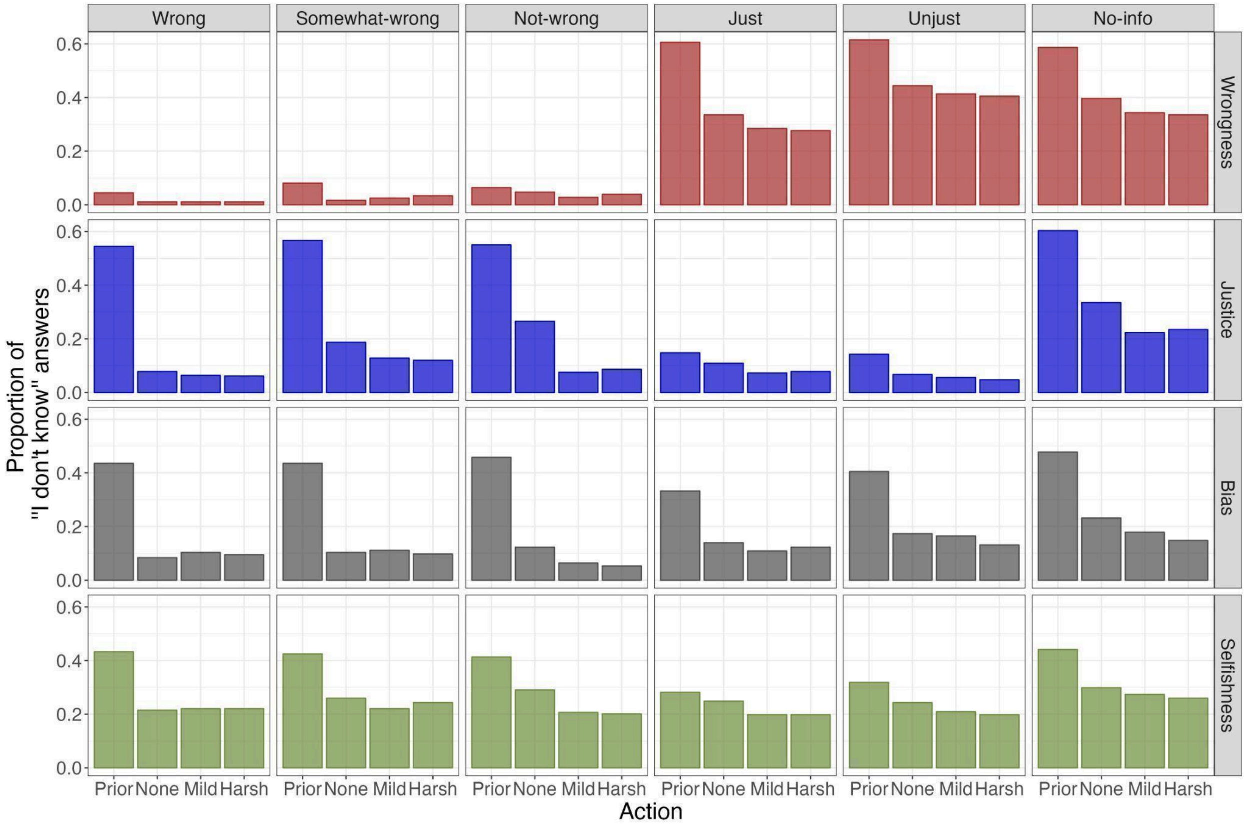

Fig. S2.4 Proportion of "I don't know. All values are equally responses" for prior and posterior beliefs.

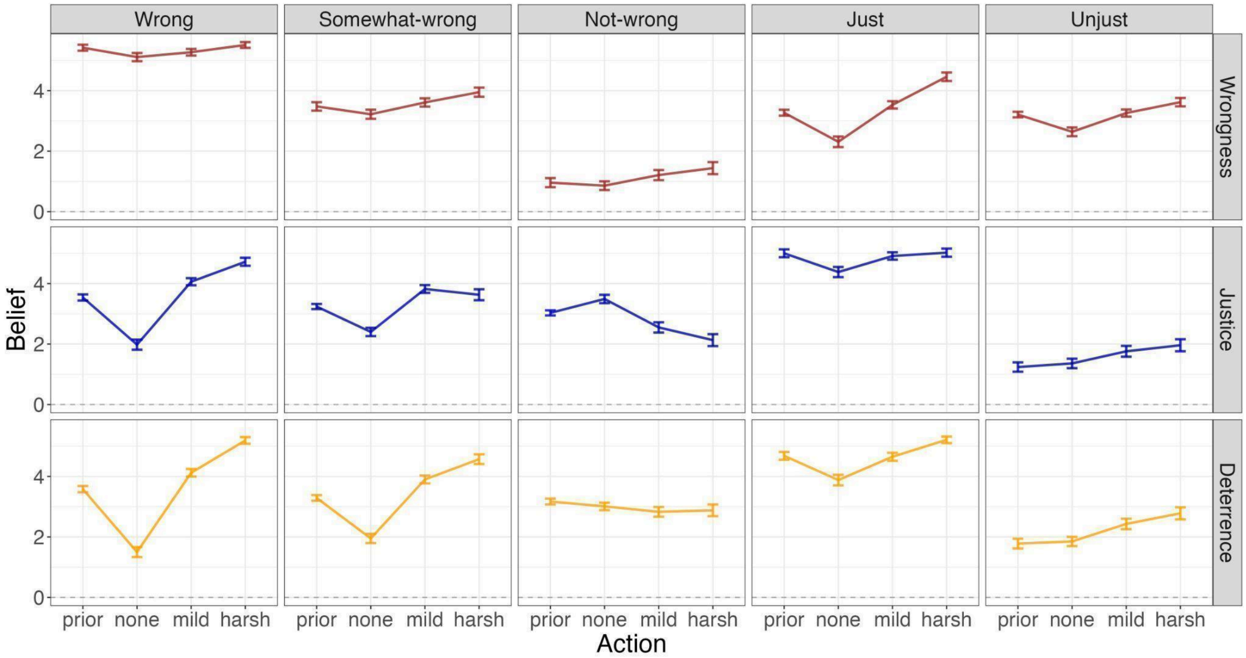

Fig. S2.5 Prior and posterior beliefs in a pilot study for study 1 (N=100; Radkani & Saxe, 2023), where we additionally measured beliefs about the authority's deterrence motive by asking "In general, how much does [Authority] care about preventing wrong behavior in this society? (0: not at all, …, 6: extremely)". The authority is judged as more motivated by deterrence when punishing harshly in response to an act that is somewhat-wrong, as opposed to justice motives that decrease. Also, in response to an act that is not wrong, none of the punitive decisions are informative about the authority's deterrence motives. This pattern of judgments suggests that observers expect authorities motivated by deterrence to monotonically increase the harshness of punishment beyond a proportional response.

Punishment failed to communicate wrongness as strongly, if the authority was described as not particularly concerned with justice. Indeed, although observers started from the same level of uncertainty, they were more likely to stay uncertain about wrongness, after observing the unfair authority's punitive decision, compared to the fair authority (main effect of "Unjust" vs "Just" on probability of "I don't know" answers: $\beta=1.372$, std=0.157, z=8.76, $p<2e-16$). Using each participant's own reported priors about the authority's justice motive in place of the experimenter label for each condition revealed that the higher participants' prior on the authority's justice motives, the more they updated their beliefs about action wrongness (interaction between justice priors and response (punish vs not-punish): $\beta=0.108$, std=0.018, t(5.5)=5.93, p=0.0014). For this analysis, we mean-centered (without scaling) the prior beliefs about the authority's justice motive within the two "Just" and "Unjust" conditions, and coded punitive responses as "None"=-1, "Mild"=0, "Harsh"=1.

Overall, authorities who punished were perceived to be only slightly more selfish than those who did not (β=0.234, std=0.045, t(62.4)=5.16, p=2.77e-06). Nevertheless, these inferences were modulated by information about wrongness of the target act as well, such that the less wrong the target act, the more punishing (compared to doing nothing) led to an increase in inferences of selfishness (*NP) (interaction between response (punish vs not-punish) and condition ("Somewhat-wrong" vs "Wrong"), β=0.436, std=0.141, t(1785)=3.09, p=0.00204; ("Not-wrong" vs "Wrong"), β=0.584, std=0.141, t(1785)=4.14, p=3.66e-05).

Model fitting and comparison.

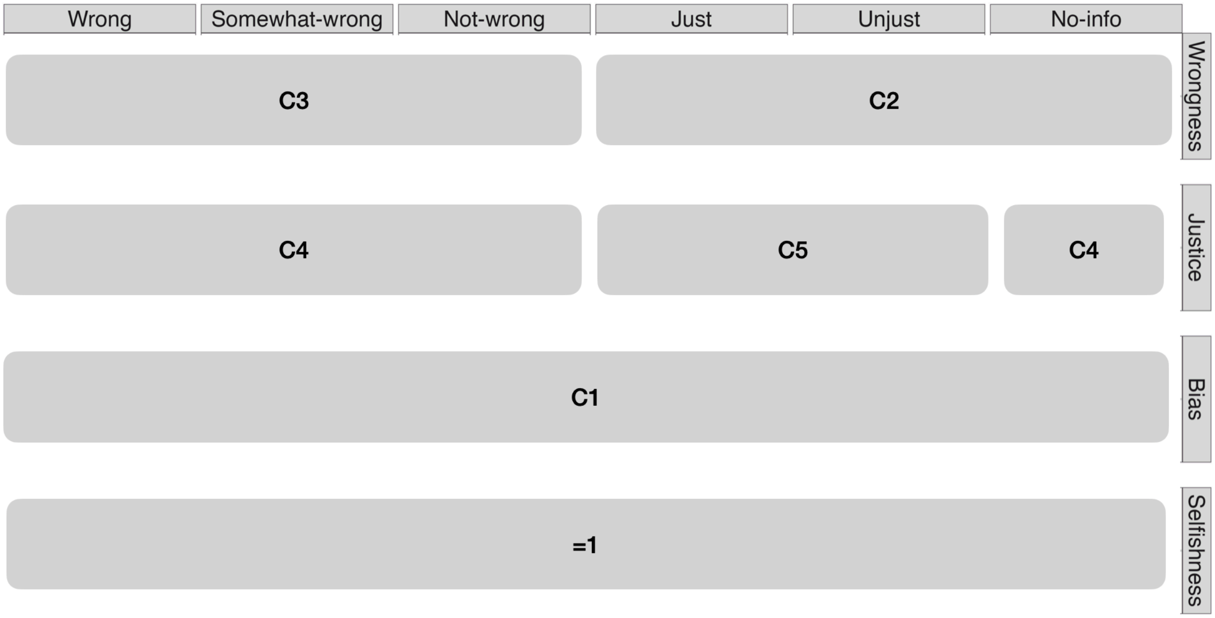

Fig. S2.6 The structure of the multiplicative constants for the 'a' and 'b' of the beta prior distributions. The prior distribution over each dependent variable was assigned a constant depending on whether there was any information provided about that variable or the prior belief was kept uncertain.

The parameters defining the concept of $U_{justice}$ were found using participants' judgments of harshness in study 1 (see Methods), as $\eta_w = -0.665$, $\eta_t = 1.016$, and were fixed in all model simulations.

Table S2.1 Best parameter fits in study 1

	Training set 1	Training set 2	Training set 3	Training set 4	Training set 5	Training set 6
α_0	0.5	0.75	0.75	0.75	0.75	0.75
α_1	Not fit in study 1					
β	13	11	11	11	13	13
γ	1.4	1.4	1.4	1.4	1.4	1.4
h_0	-0.15	-0.15	-0.15	-0.15	-0.15	-0.15
C_1	1	1	1	1	1	1
C_2	2	2	2	2	3	2
C_3	5	5	5	5	5	5
C_4	0.5	1	1	1	1	1
C_5	2	2	2	2	2	2

Note: Training set 'x' consists of all scenarios but scenario 'x'.

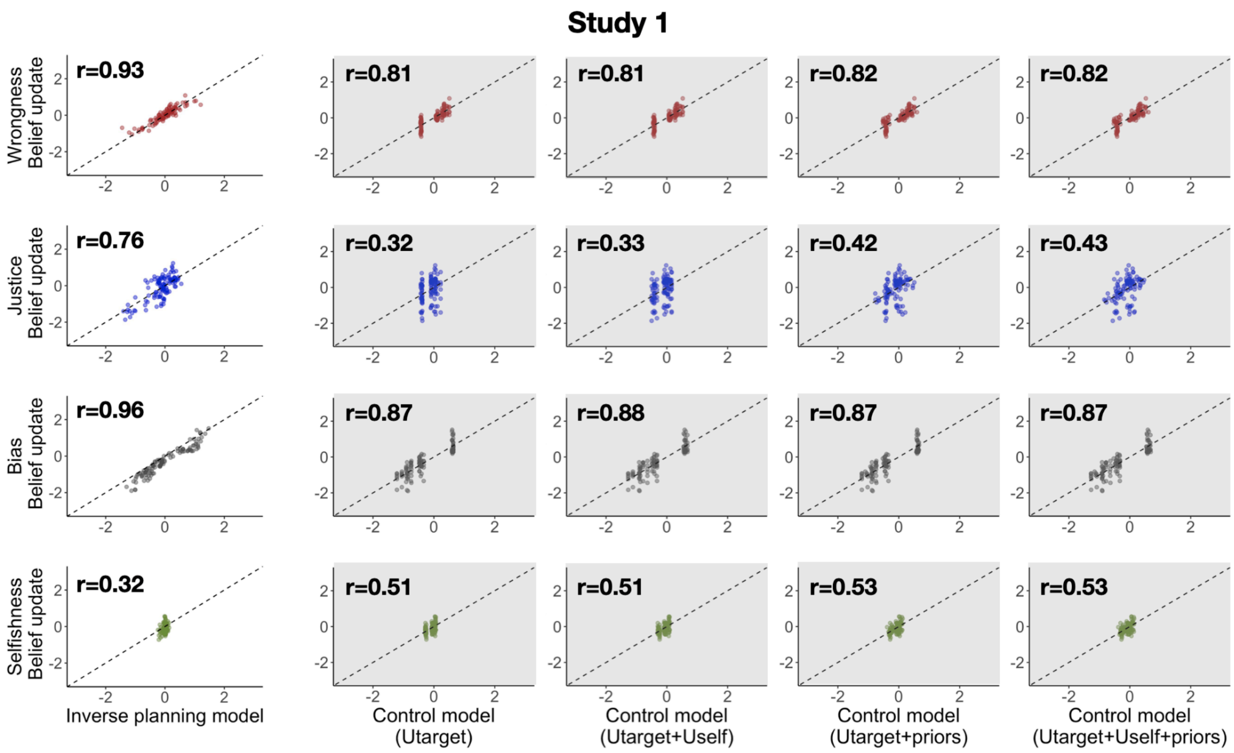

Fig. S2.7: Performance of the inverse planning and all the control models in capturing observers' judgments in study 1. The data points show the best-fitting model predictions for every scenario, condition, and response combination. For each scenario, the model was fit on the other 5 scenarios and used to make predictions on that held-out scenario. The average cross-validated r across the 6 scenarios is shown on each plot. The same procedure was used for the inverse planning and control models. The four sets of control models differ in their regressors (shown on the x-axis). The third and fourth set of control models use the average reported prior belief over each dependent variable as an additional regressor to predict the belief updates about that dependent variable, in each scenario and prior condition separately.

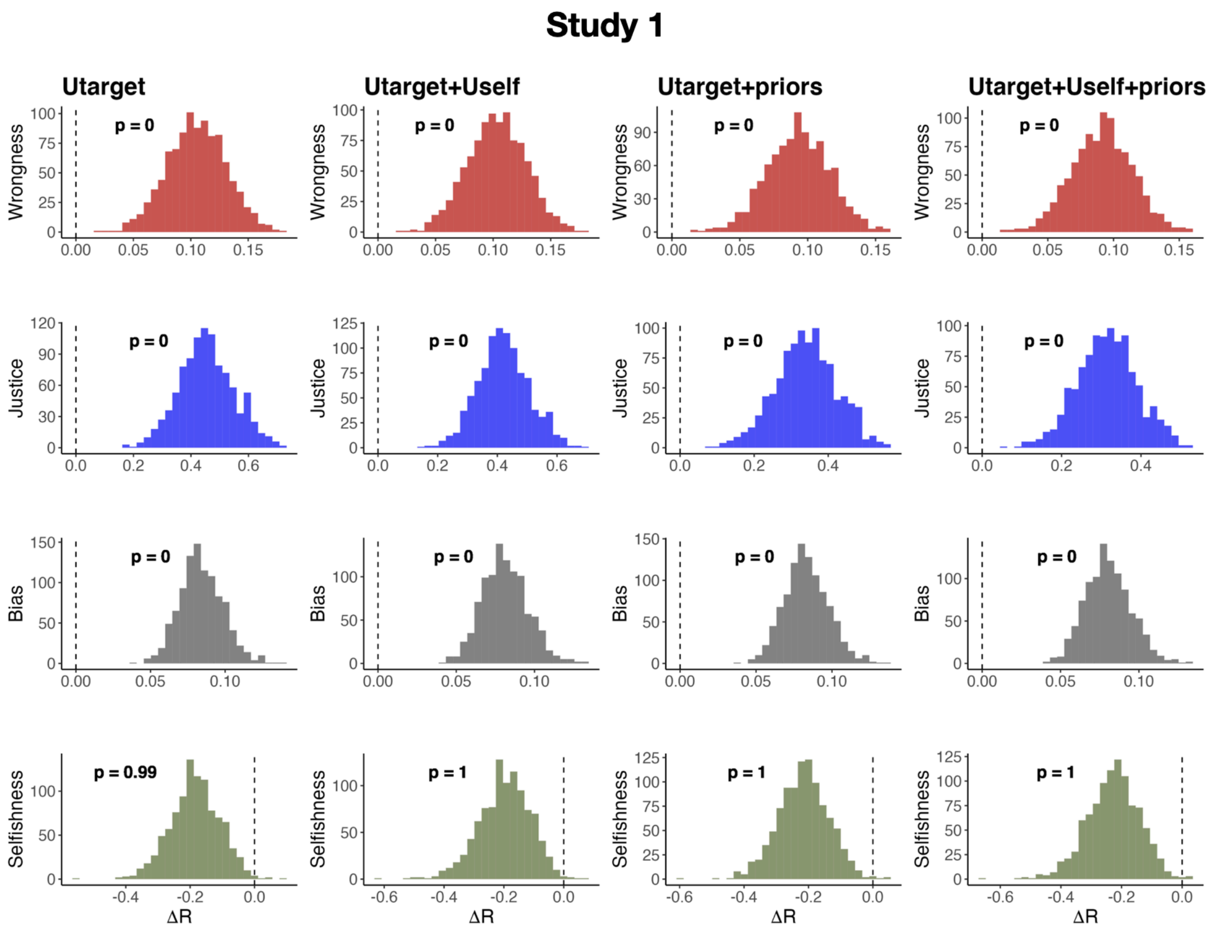

Fig. S2.8 Model comparison using bootstrapping in study 1. N=1000 with replacement from the scenario-condition-action combinations. Each plot shows the distribution of the difference between performance (i.e., r) of the inverse planning model and a control model (written on the top of the column) fit to the sampled dataset. The "p" values show the proportion of samples below zero (i.e., the sampled datasets for which the control model outperformed the inverse planning model).

3. Study 2

Materials and Design.

Allyship condition	Allyship information
**Ally**	You also hear that …<br>- version 1: [Target] is one of [Authority]'s supporters.<br>- version 2: [Authority] is allied with [Target], in competition against others for status and resources.<br>- version 3: [Target] is [Authority]'s personal ally.
**Neutral**	-
**Competitor**	You also hear that …<br>- version 1: [Target] is one of [Authority]'s opponents.<br>- version 2: [Authority] is allied with others, in competition against [Target] for status and resources.<br>- version 3: [Target] is [Authority]'s personal enemy.

Fig. S3.1 Manipulation of the authority-target relationship (i.e., allyship) in study 2. The same wording was used for all the scenarios (depending on the version, see Fig. S2.2).

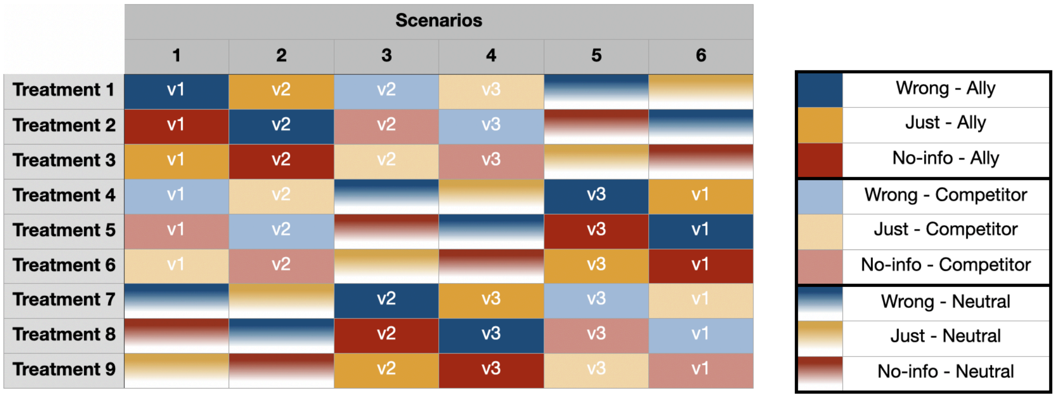

Fig. S3.2 Counterbalancing of scenarios and conditions across participants. Each participant was randomly assigned to a treatment (i.e., one row of this matrix) by the Qualtrics randomizer. The order of the scenarios were also randomized within each participant. Each scenario used the two corresponding versions of allyship manipulation for the ally and competitor conditions (e.g., scenario 1 used version 1 for both ally and competitor conditions), so that after pooling the data across treatments, the different prior conditions of each scenario would be more comparable. Each participant saw all three allyship conditions, across two out of three prior conditions from study 1 (i.e., "Wrong", "Just", "No-info").

Manipulation check pilot.

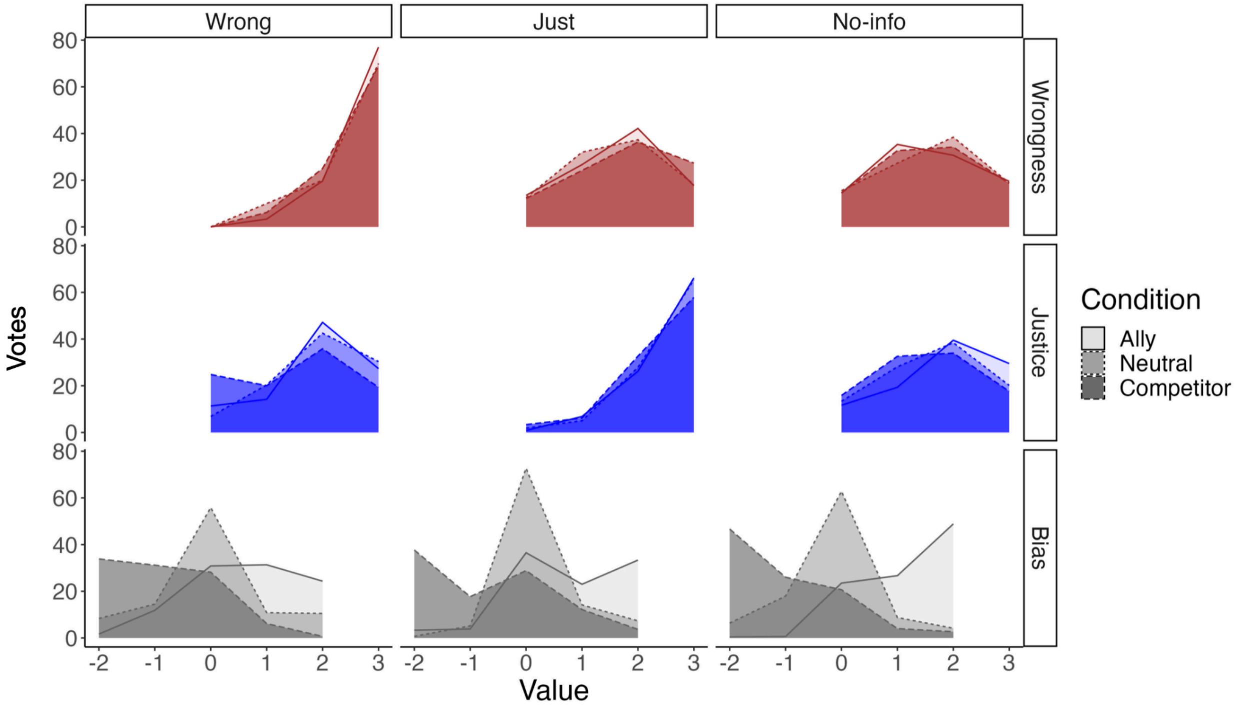

Fig. S3.3 Participants' prior beliefs in the manipulation check pilot study (N=30). The pilot used the same design as study 2, except that we measured both the value and uncertainty of participants' priors by asking the participants to distribute 100 votes on the relevant scale for each question. The plot shows the average of all participants' belief distributions.

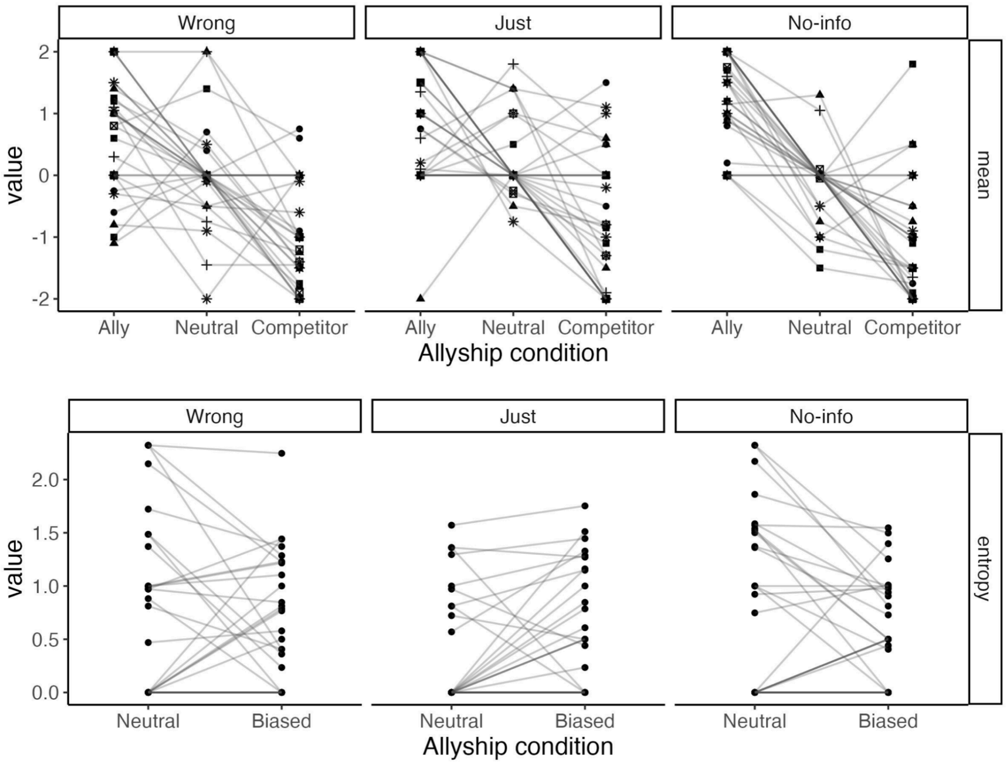

Fig. S3.4 Participants' prior beliefs in the manipulation check pilot study (N=30). The plot shows the mean (upper panel) and the entropy (lower panel) of participants' belief distributions, within each prior condition separately. Each participants' belief statistics across different prior conditions is connected with a line. For entropy, the average of "Ally" and "Competitor" conditions for each participant is reported as the "Biased" condition, in order to compare the entropy of beliefs in conditions where some information is provided about the authority-target relationship versus conditions where no information is provided (i.e., "Neutral").

Supplementary results.

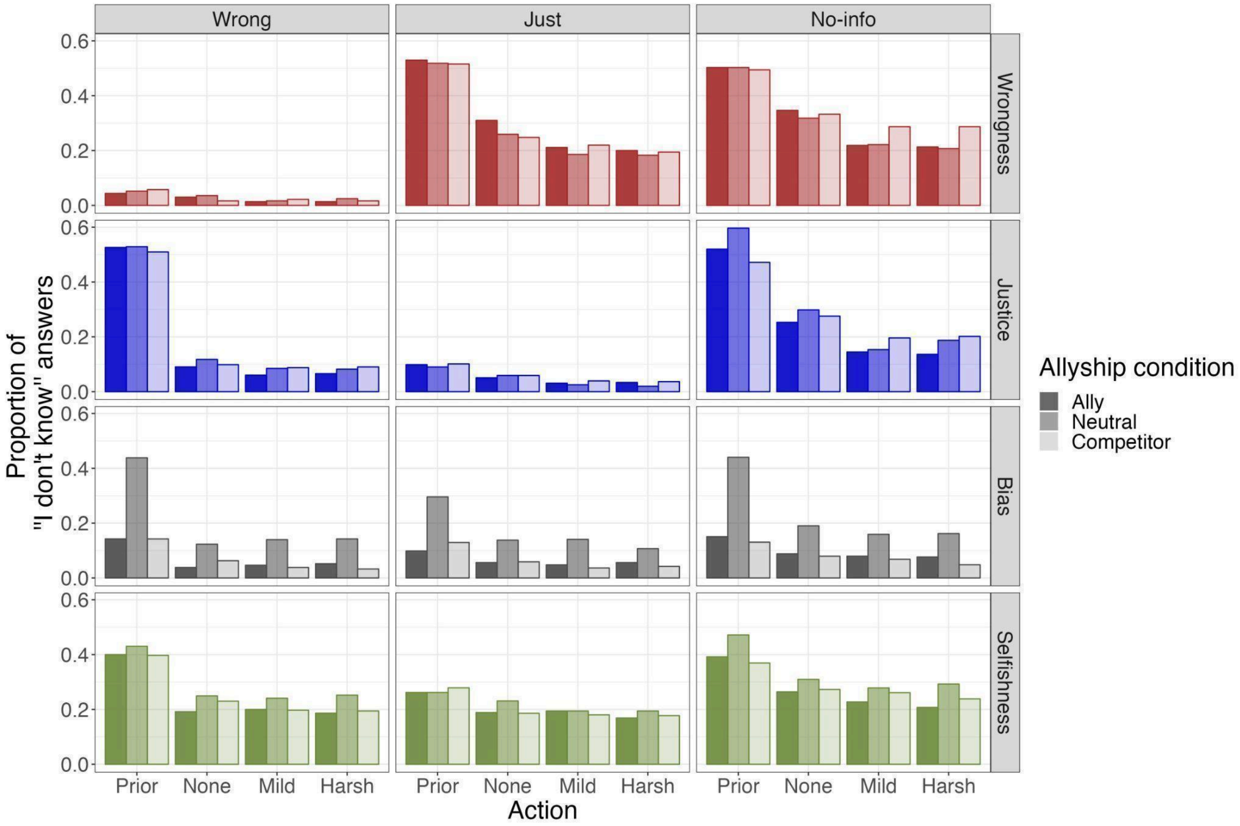

Fig. S3.5 Proportion of “I don’t know. All values are equally” responses for prior and posterior beliefs.

Replicating study 1.

We replicated the key findings from study 1. In study 2 (Fig. 4A), punishment communicated about wrongness effectively when performed by a just authority in response to a target act about which the observer was uncertain (main effect of punish vs not-punish: $\beta=1.463$, std=0.101, $t(7.3)=14.44$, $p=1.27e-06$), especially the more the authority is believed to be motivated by justice (interaction between response and participants’ own judgments of the authority’s justice motive: $\beta=0.297$, std=0.074, $t(685.9)=4.02$, $p=6.46e-05$). On the other hand, observers used punishment to learn about the authority’s justice motive when they were certain about the target act but uncertain about the authority (main effect of punish vs not-punish: $\beta=2.196$, std=0.114, $t(8.9)=19.25$, $p=1.38e-08$).

Inferences about wrongness.

In the “Just” and “No-info” conditions of study 2, prior beliefs about wrongness were uncertain, and varying prior beliefs about the authority’s bias modulated inferences about wrongness (Fig. 4B). Observers learned more about wrongness from punishment by an ally, or

doing nothing by a competitor, responses that are unexpected given the authority-target relationship (interaction; marginally in “Just” condition: β=0.176, std=0.073, t(5.5)=2.40, p=0.057; significantly in “No-info” condition: β=0.269, std=0.075, t(13.2)=3.61, p=0.0031). The same result mostly held when using each participant's own reported priors about the authority’s bias in place of the experimenter label for the authority-target relationship (marginally in the “Just” condition: β=0.142, std=0.0066, t(6)=2.14, p=0.076; significantly in the “No-info” condition: β=0.136, std=0.039, t(19.5)=3.43, p=0.0028).

The inverse planning model (average r=0.91) outperformed all the control models (average r=0.83, 0.84, 0.84, 0.84; ps<0.001)

Inferences about the authority’s justice motive and bias.

In the “Wrong” and “No-info” conditions, prior beliefs about the authority’s justice motives were uncertain, and varying prior beliefs about the authority’s bias modulated inferences about justice motives. Observers more strongly updated their beliefs about the justice motive of an ally versus a competitor, both when using experimenter labels (interaction between punitive response and the authority-target relationship; “Wrong” condition: β=0.233, std=0.073, t(4.5)=3.21, p=0.0278 (*NP); “No-info” condition: β=0.327, std=0.075, t(5.5)=4.39, p=0.0058) and participant’s own priors (interaction between punitive response and prior on the authority’s bias; “Wrong” condition: β=0.148, std=0.052, t(5.5)=2.82, p=0.033 (*NP); “No-info” condition: β=0.225, std=0.041, t(8)=5.54, p=5.52e-04).

For inferences about justice, the performance of the inverse planning model (average r=0.69) was neither better nor worse than the control models without ‘prior’ as regressor (average r=0.75, 0.76; ps=0.89, 0.91) and was worse than the control models with ‘prior’ as regressor (average r=0.82, 0.83; ps>0.999).

For inferences about bias, the inverse planning model (average r=0.91) outperformed the control models without ‘prior’ (average r=0.84, 0.84; ps<0.0010), but was neither better nor worse than the control models with ‘prior’ as regressor (average r=0.92, 0.91; ps=0.79, 0.34).

Model comparison.

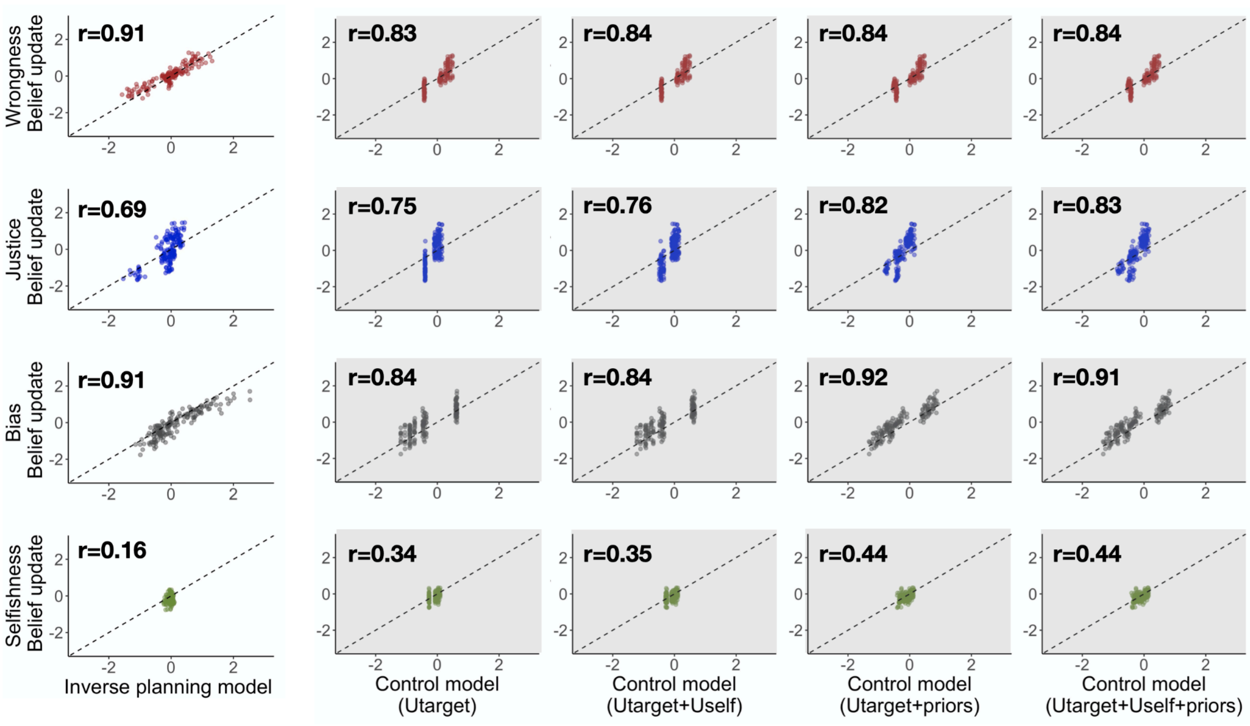

Fig. S3.6 Performance of the inverse planning and all the control models fit to study 1 data, in capturing observers' judgments in study 2. The average r across the 6 scenarios is shown on each plot. The same procedure was used for the inverse planning and control models.

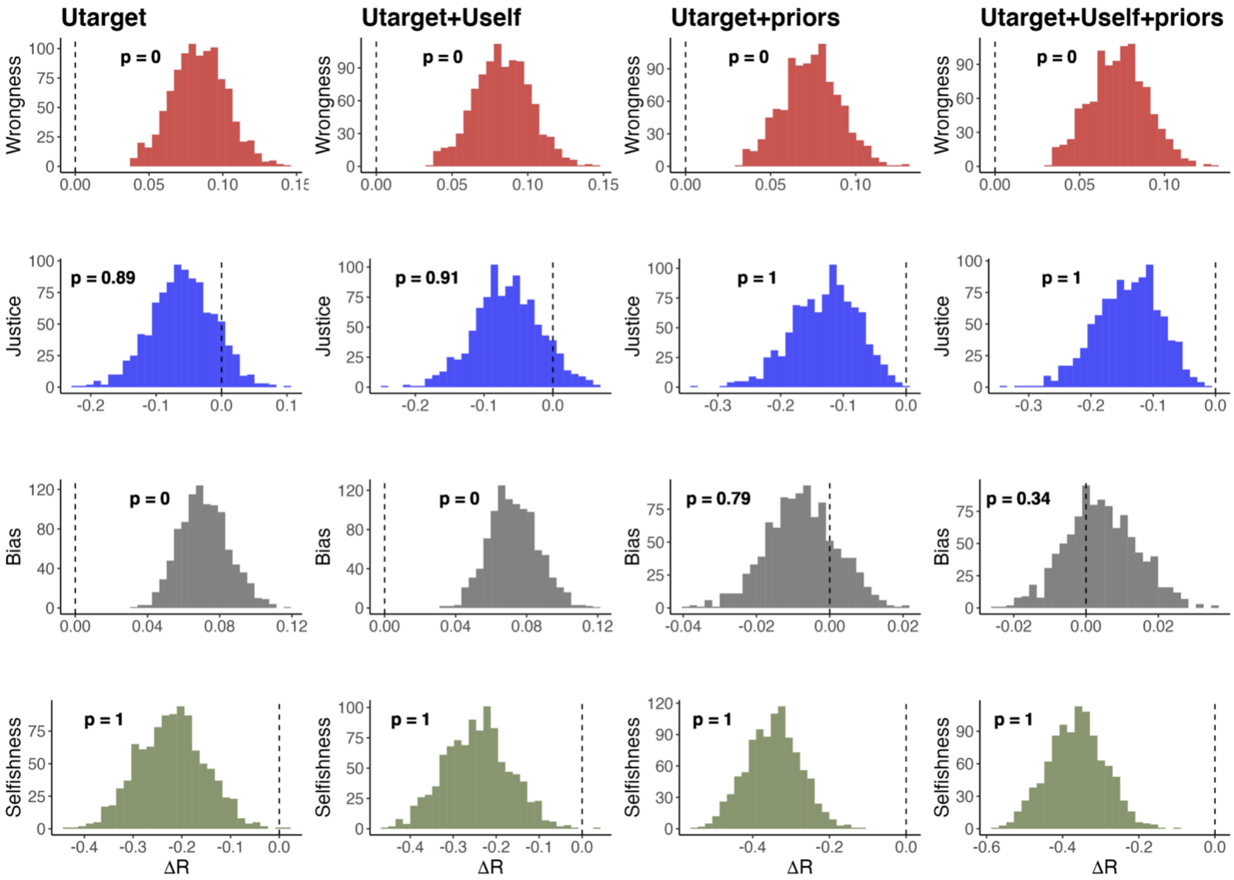

Fig. S3.7 Model comparison using bootstrapping in study 2. N=1000 with replacement from the scenario-condition-action combinations. Each plot shows the distribution of the difference between the performance (i.e., r) of the inverse planning model and a control model (written on the top of the column), all fit in study 1. The "p" values show the proportion of samples below zero (i.e., the sampled datasets for which the control model outperformed the inverse planning model).

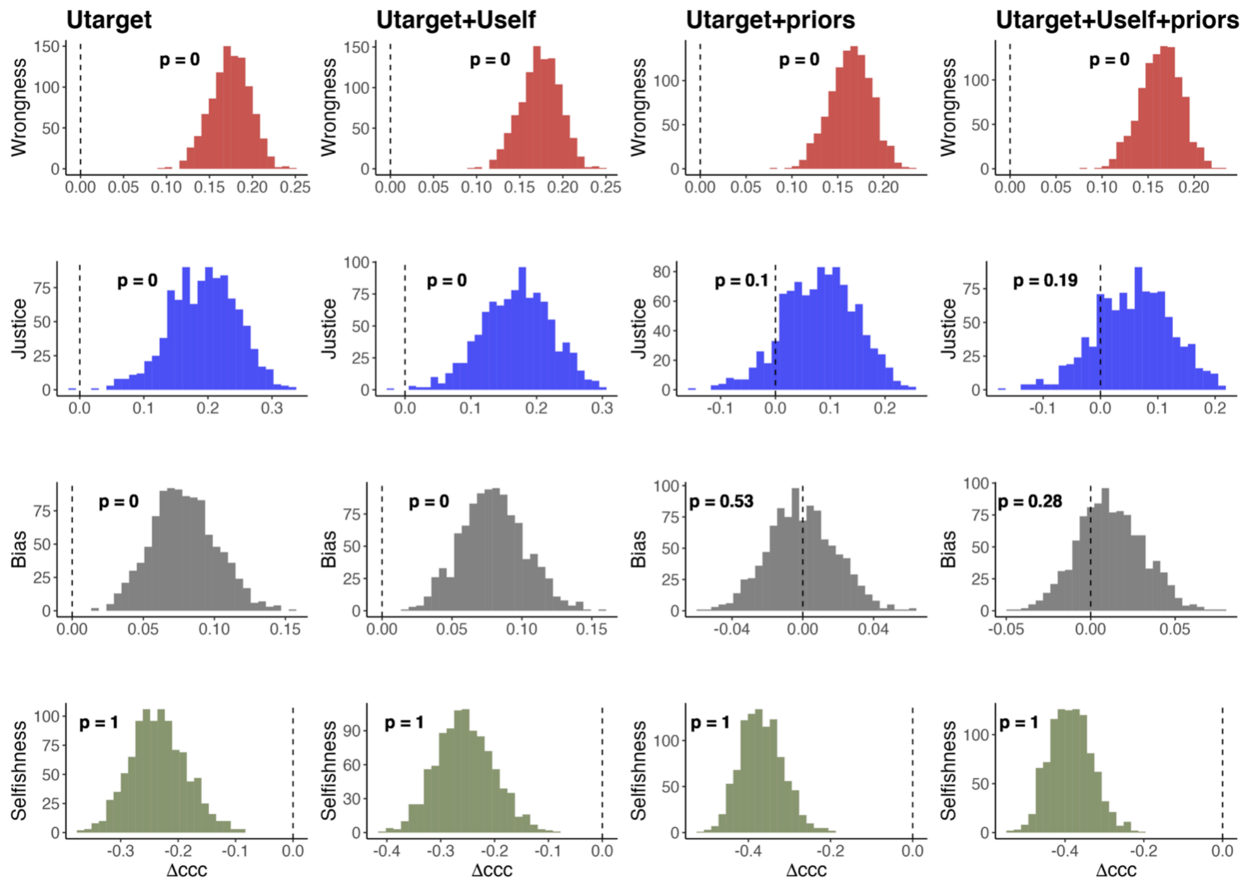

Fig. S3.8 Model comparison results in study 2, with Concordance Correlation Coefficient (CCC) as the performance measure.

4. Study 3

Materials and Design.

Scenario	Direct consequences of punishment	Direct consequences of doing nothing
S1	Cost: If Paji takes away Tudo's jats, Paji will lose a large amount of their own jats as well.<br>Benefit: If Paji takes away Tudo's jats, Paji can keep a large amount of the jats for themself. Doing nothing has no direct costs or benefits for Paji.	Doing nothing has no direct costs or benefits for [authority].
S2	Cost: By not allowing Kiva to go to the mot, Didi has to give up time at the mot to watch Kiva. Doing nothing has no direct costs or benefits for Didi.<br>Benefit: By not allowing Kiva to go to the mot, Didi gets extra time at the mot. Doing nothing has no direct costs or benefits for Didi.	
S3	Cost: By forcing Buba to clean the deb, Fadi has to spend time painstakingly watching Buba cleaning. Doing nothing has no direct costs or benefits for Fadi.<br>Benefit: By forcing Buba to clean the deb, Fadi gets time off from cleaning the deb. Doing nothing has no direct costs or benefits for Fadi.	
S4	Cost: By taking away the voca, Maki will also be unable to use the voca in that period. Doing nothing has no direct costs or benefits for Maki.<br>Benefit: By taking away the voca, Maki will be able to have the pleasure of using the voca much more often in that period. Doing nothing has no direct costs or benefits for Maki.	
S5	Cost: By forcing Lula to wear xoops, Vivi will have to spend a lot of time doing all the work assembling the xoops each morning and disassembling the xoops each evening for that period. Doing nothing has no direct costs or benefits for Vivi.<br>Benefit: By forcing Lula to wear xoops, Vivi will be immune from wearing xoops for that period. Doing nothing has no direct costs or benefits for Vivi.	
S6	Cost: By forcing Zuba to contribute, Nadi has to contribute one barrel of food to community resources as well. Doing nothing has no direct costs or benefits for Nadi.<br>Benefit: By forcing Zuba to contribute, Nadi will be exempt from contributing a whole barrel of food next month. Doing nothing has no direct costs or benefits for Nadi.	

Fig. S4.1 Manipulation of the direct consequences of punishment for the authority in study 3. Doing nothing was always described as having no consequences. For the "No-consequence" condition, the same wording as "doing nothing" was used, for example, "Either doing nothing or taking away Tudo's jats has no direct costs or benefits for Paji".

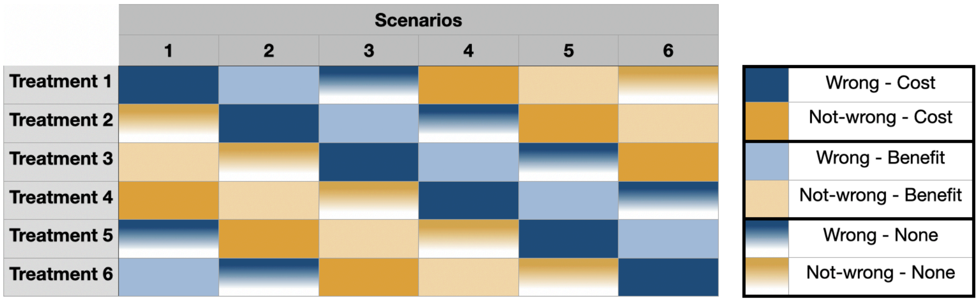

Fig. S4.2 Counterbalancing of scenarios and conditions across participants using a latin square design. Each participant was randomly assigned to a treatment (i.e., one row of this matrix) by the Qualtrics randomizer. The order of the scenarios were also randomized within each participant.

Manipulation check pilot.

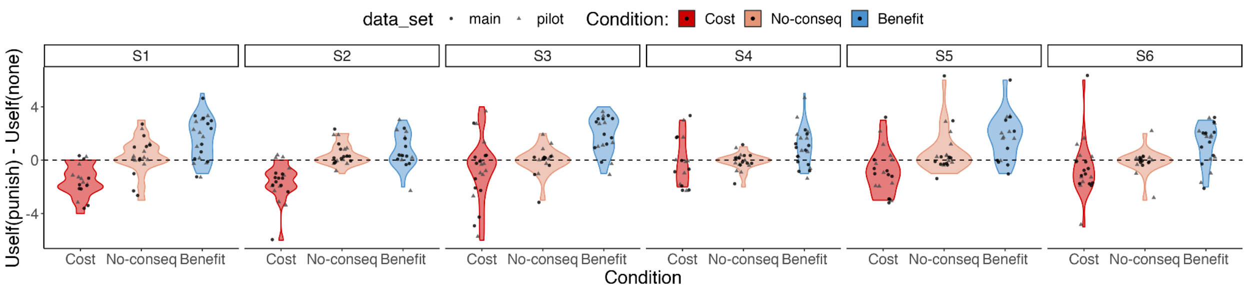

Fig. S4.3 Participants' judgments of the direct consequences of punishment versus doing nothing for the authority, in both a manipulation check pilot (N=30; only asking for U_{self} judgments without the other DVs), and a main pilot for study 3 (N=30; using the exact same DVs as study 3).

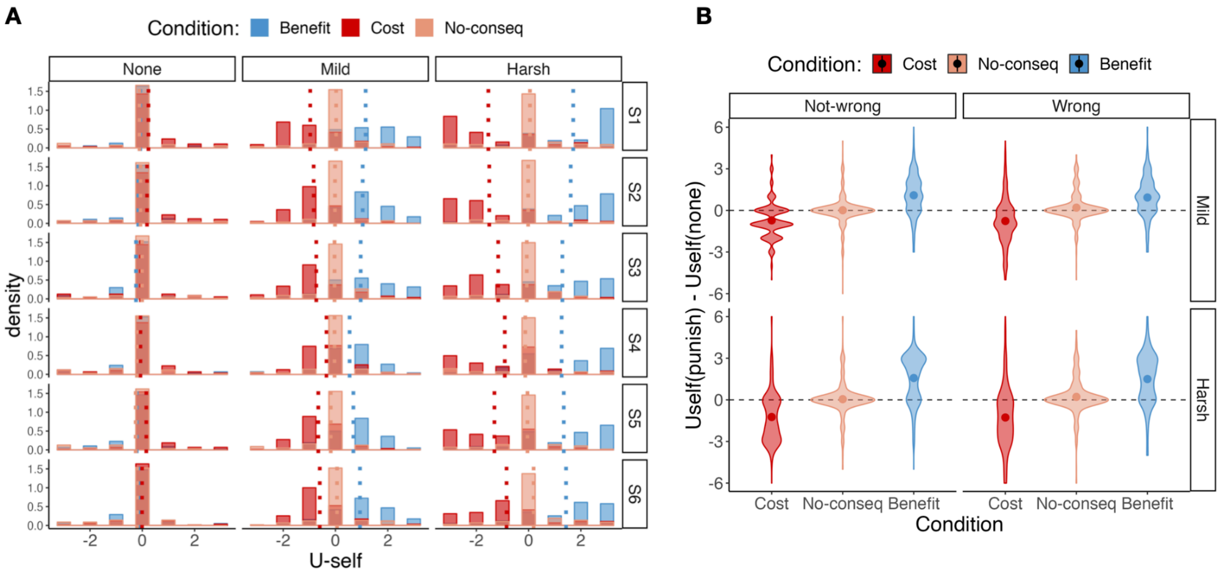

Fig. S4.4: Distribution of participants' judgments of the direct consequences of each punitive response in study 3. A) across "Not-wrong" and "Wrong" prior conditions combined. The rows correspond to each scenario, the columns correspond to each punitive response, and the color of the bars determine the experimental condition for the self-directed consequences of punishment (i.e., "Cost", "No-consequence", "Benefit"). Doing nothing (i.e., "None") was always described as having no consequences; B) across all scenarios combined. The columns correspond to the prior conditions ("Not-wrong", "Wrong"), the colors and the x-axis show the experimental condition for the self-directed consequences of punishment. Y-axis shows the difference between a participant's judgment of the self-directed

consequences of either mild (top row) or harsh (bottom row) punishment versus doing nothing.

Supplementary results.

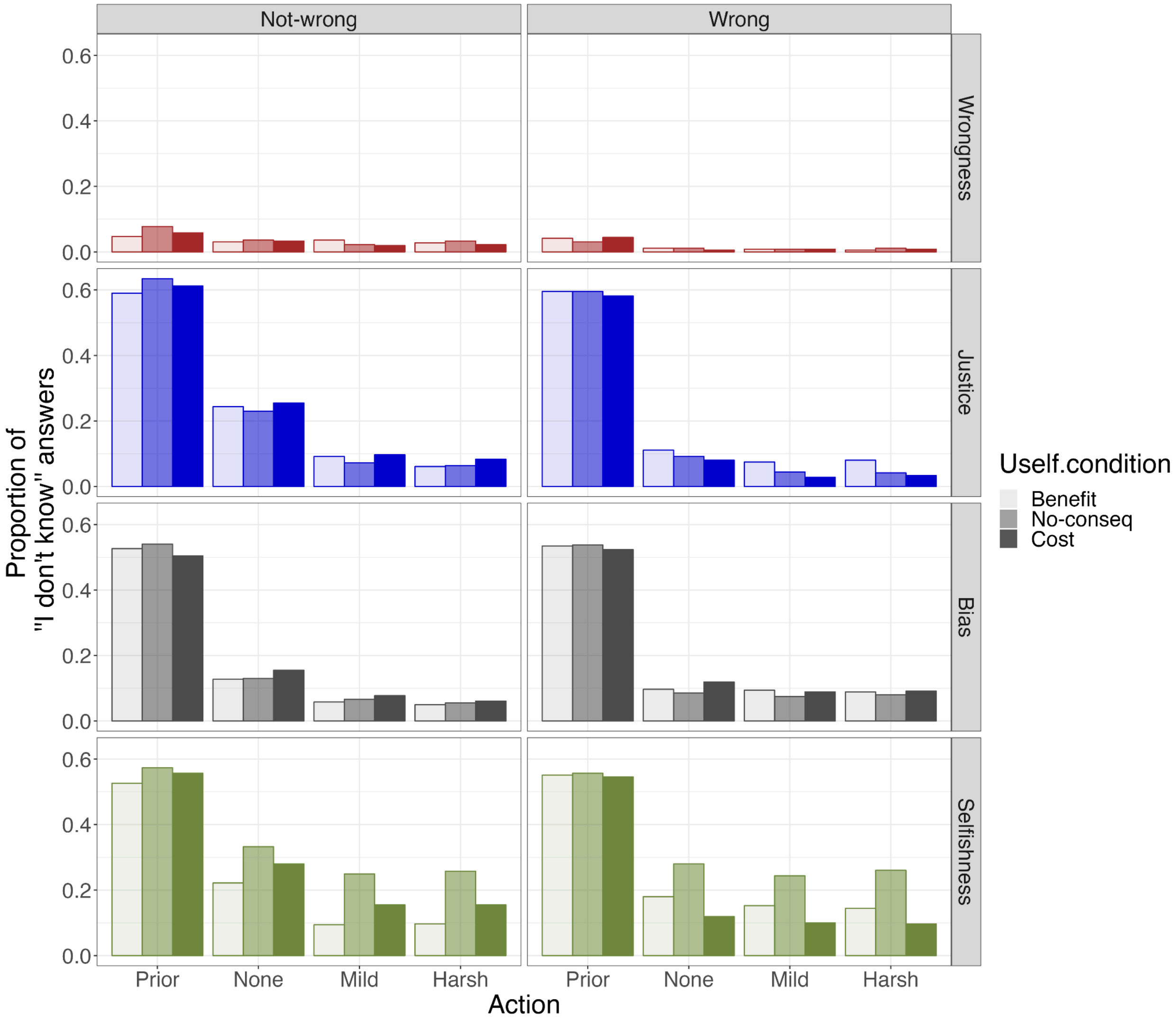

Fig. S4.5 Proportion of "I don't know. All values are equally" responses for prior and posterior beliefs.

We replicated the key findings from study 1. The proportionality of the punitive choice to the wrongness of the target act (i.e., punishing a more wrong act and doing nothing in response to a less wrong act) was the main determinant of judgements about the authority's justice motive (interaction between response (punish vs not-punish) and condition ("Not-wrong" vs "Wrong"), β=-4.269, std=0.174, t(11.8)=-24.57, p=1.59e-11; main effect of auxiliary variable "unjust": β=-2.134, std=0.093, t(17.3)=-22.99, p=1.94e-14), and the authority's bias (main effect of response (punish vs not-punish): β=-1.846, std=0.0945, t(5.1)=-19.53, p=5.35e-06;

interaction between response and auxiliary variable "unjust": β=-2.250, std=0.112, t(713.6)=-20.02, p<2e-16).

Inferences about the authority's justice motive and bias.
The self-directed consequences of punishment (relative to doing nothing) modulated inferences about the authority's justice motive, beyond the effects of punitive response and its proportionality. First, on average the more the punitive choice was against the authority's self-interest, the more the authority was judged to be motivated by justice (*NP), both using experimenter labels (main effect of 'against-self-interest': β=0.136, std=0.025, t(298.8)=5.51, p=7.81e-08) and participants' own priors (β=0.054, std=0.014, t(13)=3.92, p=1.75e-03). Second, observers more strongly updated their beliefs about the authority's justice motive as a function of proportionality of the response, the more the punitive choice was against the authority's self-interest and thereby unexpected (main effect of 'unjust': β=-0.411, std=0.014, t(325.6)=-30.41, p<2e-16; interaction of 'against-self-interest' and 'unjust': β=-0.0097, std=0.0044, t(154.1)=-2.18, p=0.0305). The interaction terms did not reach significance when using experimenter labels (interaction of 'against-self-interest' and 'unjust': β=-0.003, std=0.025, t(182.2)=-0.14, p=0.89).

In capturing belief updates about justice, the inverse planning model (average r=0.82) outperformed all the control models (average r=0.16, -0.07, 0.14, -0.08; ps<0.001).

Inferences about the authority's bias.
The self-directed consequences of punishment (relative to doing nothing) modulated inferences about the authority's bias (Fig. 5C & 5F), beyond the effects of punitive response and its proportionality. Observers more strongly updated their beliefs about the authority's bias as a function of proportionality of the response, the more the punitive choice was against the authority's self-interest and thereby unexpected (interaction of action and 'unjust': β =-0.441, std=0.012, t(3739)=-36.22, p<2e-16; interaction of 'against-self-interest', action and 'unjust': β=-0.0169, std=0.007, t(3502)=-2.45, p-value=0.0145). The interaction terms did not reach significance when using experimenter labels (interaction of 'against-self-interest', action and 'unjust' for bias: β=-0.137, std=0.087, t(3959)=-1.57, p=0.116).

The inverse planning model (average r=0.91) outperformed the control models (average r=0.84, 0.57, 0.84, 0.58; ps<0.001).

Inferences about the authority's selfishness.
The more the punitive choice (relative to the unchosen alternative) was against the authority's self-interest, the less selfish the authority appeared, both when operationalized via

experimenter labels (main effect of 'against-self-interest': β=-0.575, std=0.063, t(6.3)=-9.14, p=7.55e-05) and via individual participants' self-reported judgments of the consequences (β =-0.319, std=0.022, t(9)=-14.40, p=1.56e-07).

However, attributions of selfishness were also modulated by the perceived proportionality of the punitive response. First, the more the punitive choice was unjust, the more the authority was judged to be selfish (**NP;* main effect of 'unjust', experimenter labels: β=0.295, std=0.081, t(5.1)=3.65, p=0.014; participants' own priors: β=0.062, std=0.011, t(7.4)=5.65, p=6.2e-04 (*NP)). Second, observers more strongly updated their beliefs about the authority's selfishness as a function of the self-consequences of the response being against the authority's self-interest, the more the punitive choice was unjust and thereby unexpected (interaction of 'unjust' and 'against-self-interest', experimenter labels: β=-0.281, std=0.078, t(5.4)=-3.59, p=0.014; participants' own priors: β=-0.041, std=0.006, t(7.9)=-6.83, p=1.38e-04).

The inverse planning model (average r=0.72) outperformed all the control models (average r=0.18, -0.65, 0.17, -0.63; ps<0.001).

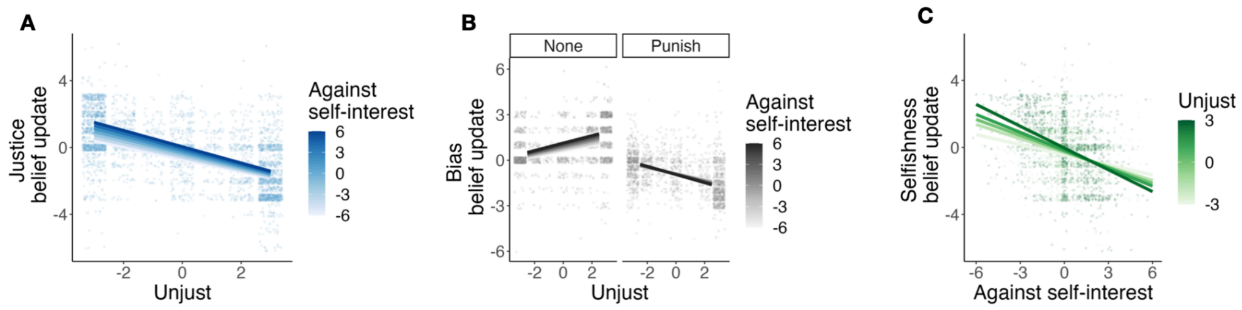

Fig. S4.6: Belief updates in study 3 as a function of participants' own judgments. Belief updates about the authority's motives as a function of auxiliary variables 'against-self-interest' and 'unjust' defined using individual participants' own priors about wrongness and their judgments of response consequences for the authority (see "Methods" for the definition of variables).

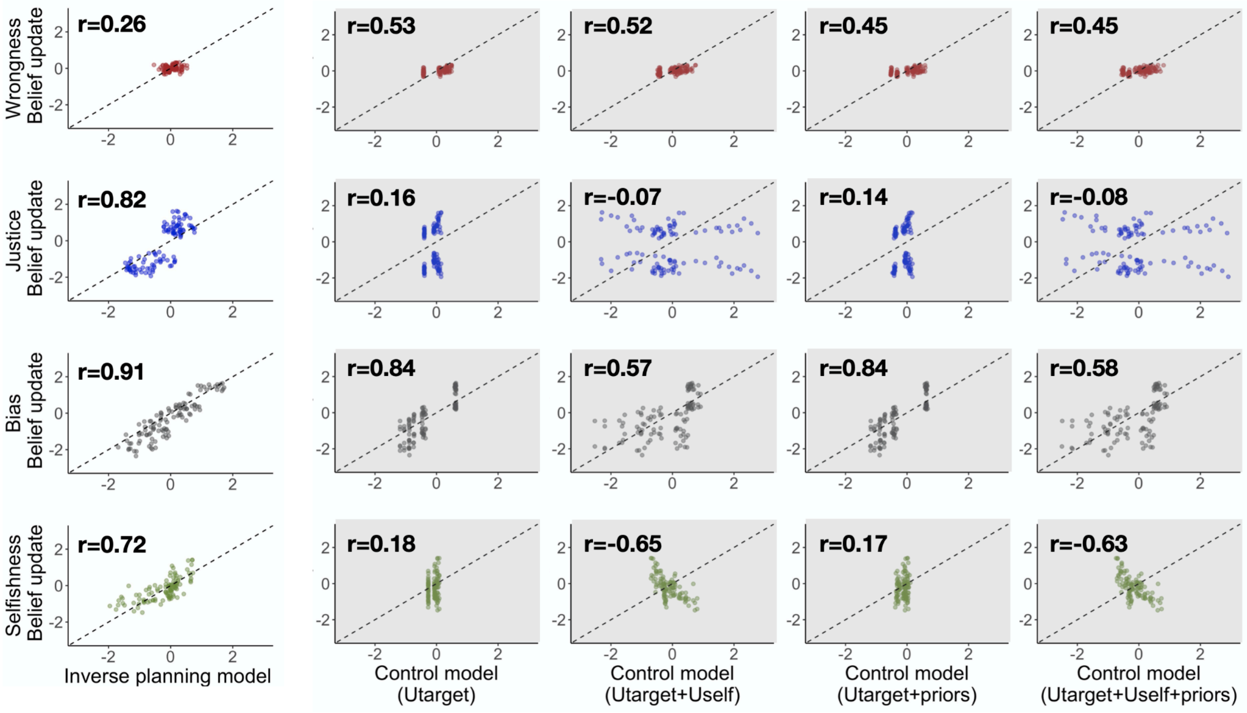

Fig. S4.7 Performance of the inverse planning and all the control models fit to study 1 data, in capturing observers' judgments in study 3. The average r across the 6 scenarios is shown on each plot. The same procedure was used for the inverse planning and control models.

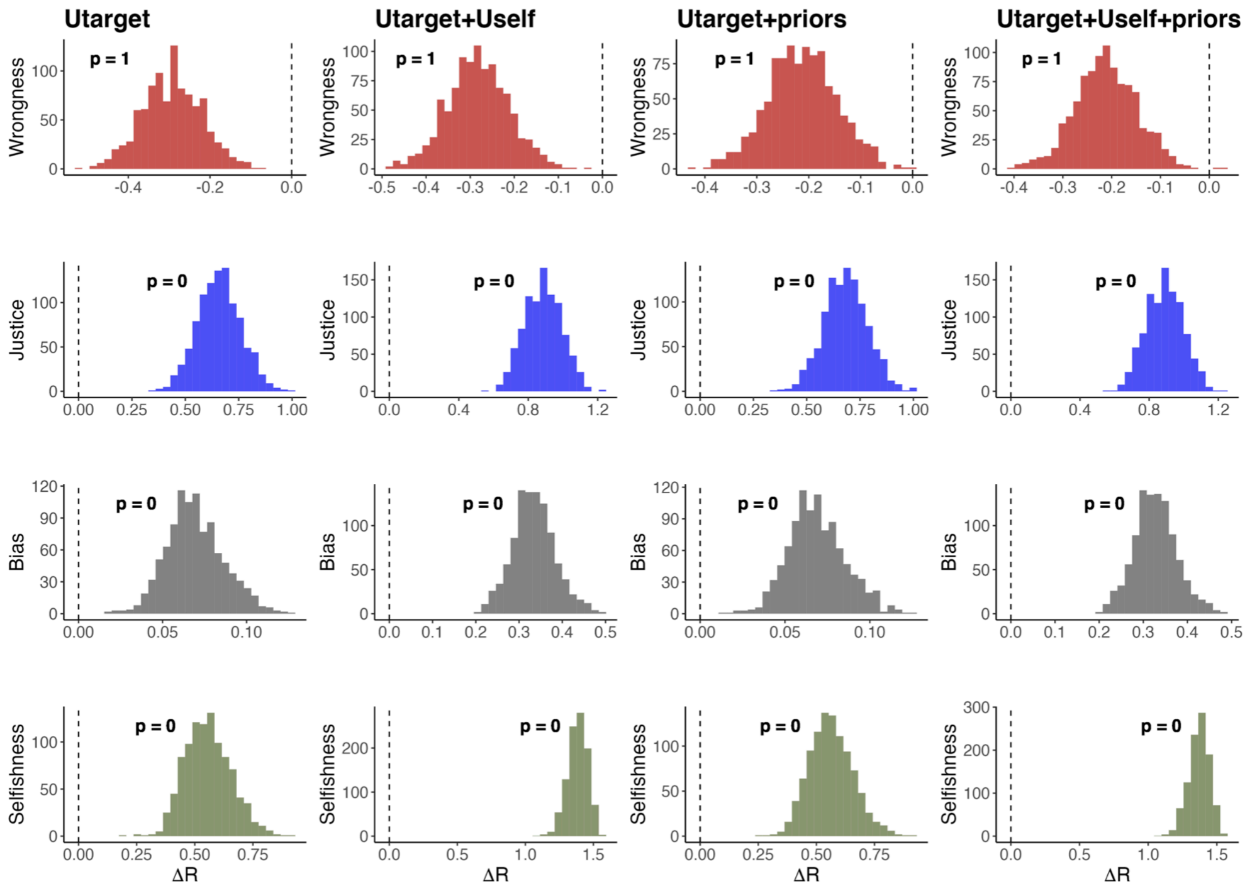

Fig. S4.8 Model comparison using bootstrapping in study 3. N=1000 with replacement from the scenario-condition-action combinations. Each plot shows the distribution of the difference between the performance (i.e., r) of the inverse planning model and a control model (written on the top of the column), all fit in study 1. The "p" values show the proportion of samples below zero (i.e., the sampled datasets for which the control model outperformed the inverse planning model).

5. Study 2&3

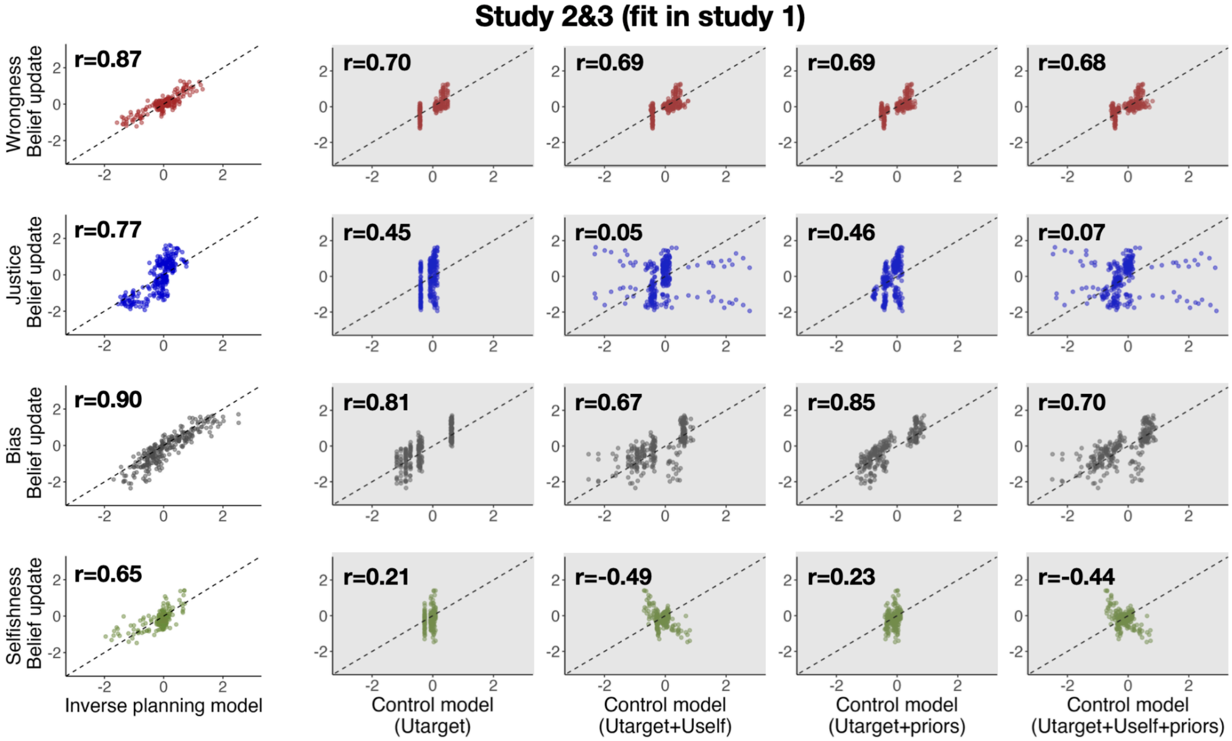

Fig. S5.1 Performance of the inverse planning and all the control models in capturing observers' judgments in study 2 and study 3 combined. The data points show the model predictions for every scenario, condition, and response combination in studies 2 and 3. The inverse planning model and the control models were fit only to study 1 data. The average r across the 6 scenarios is shown on each plot.

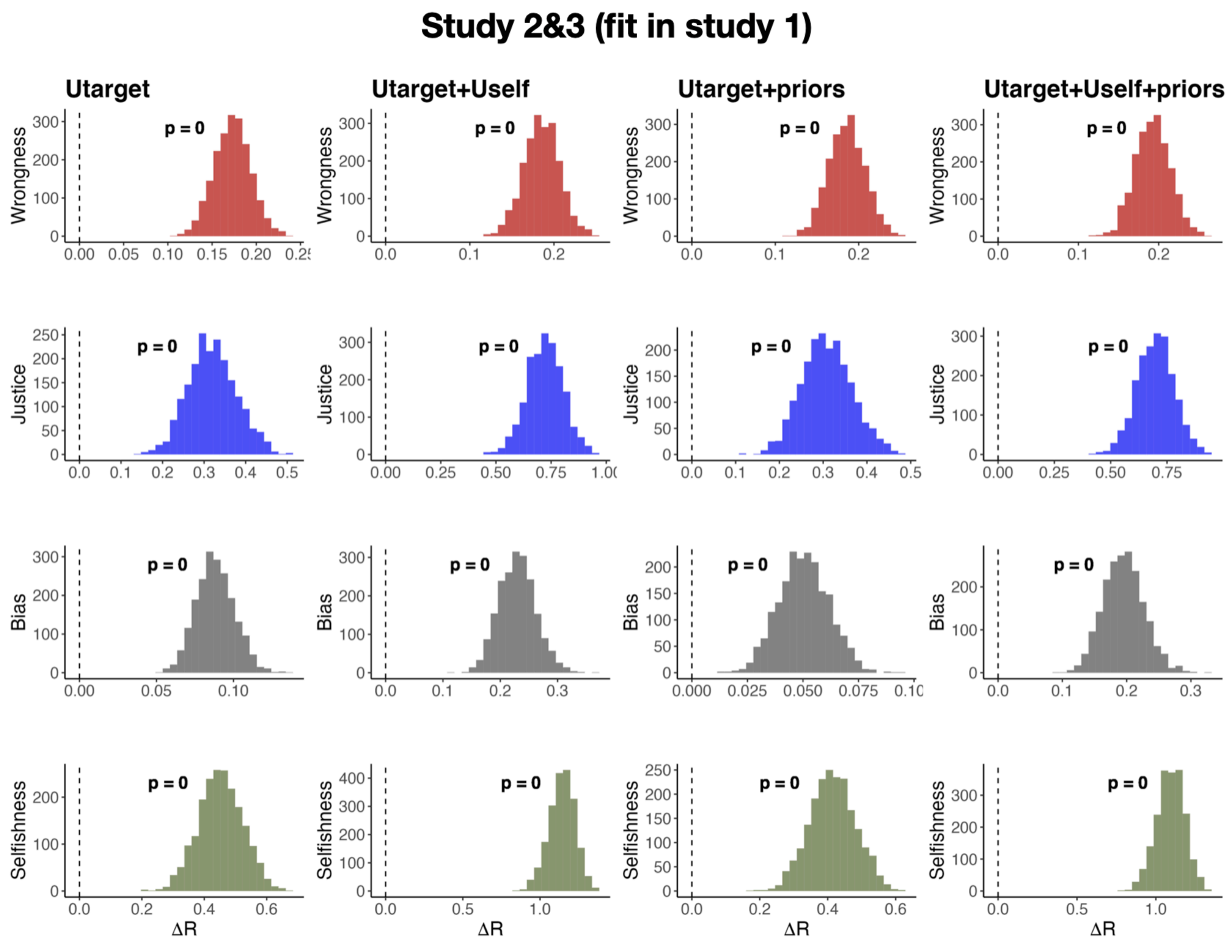

Fig. S5.2 Model comparison using bootstrapping in study 2 and study 3 combined. N=2000 with replacement from the scenario-condition-action combinations. Each plot shows the distribution of the difference between performance (i.e., r) of the inverse planning model and a control model (written on the top of the column) fit to the sampled dataset. The “p” values show the proportion of samples below zero (i.e., the sampled datasets for which the control model outperformed the inverse planning model).

6. Study 4

Materials and design.

In study 4, the scenario followed a similar structure as study 1-3 (Fig 2 in main text), introducing an authority, a novel target act and a means of punishment. We used the authority (i.e., Paji), the target act (i.e., daxing) and the means of punishment (i.e., taking away jats) from scenario 1 of studies 1-3. In the "Wrong" and "Not-wrong" conditions no information was provided about Paji, but the participants read that "You hear that daxing is fun but it is very harmful to other people" and "You hear that daxing is fun and it is not harmful to other people", respectively. In the "Just" condition, participants read that "You hear that Paji has a strong sense of justice and tries to make sure everyone is treated fairly" and "You don't know anything about daxing". The scenario continued by saying "During the week, Paji goes around and checks how everyone else is acting. After all, some people may be harming others or acting selfishly, and in response, Paji could either do nothing, take away half of their jats, or take away all of their jats".

After reading this information we measured participants' prior beliefs about wrongness of daxing and the authority's justice motives, using the same DVs as studies 1-3. The remainder of the experiment consisted of four trials; in each trial, we provided an observation of harsh punishment (e.g., On Monday, Paji found Tudo daxing. Paji decided to take away all of Tudo's jats.) followed by the same two DVs to measure wrongness of daxing and Paji's justice motives. We used Tudo, Kiva, Buba, Sudo as the name of the targets being punished on Monday, Tuesday, Wednesday and Thursday. As exploration, we also included a fifth trial where Paji decides to do nothing (i.e., "On Friday, Paji found Lula daxing. Paji decided to do nothing"); we did not include the results of this trial in any of our analyses.

We pre-registered and ran two versions of study 4 (version 1: N=156, version 2: N=165) which only differed in one sentence in the scenario. In version 1 (pre-registration: https://osf.io/6pg8e), we found that about 20% of participants in the "Not-wrong" condition replied "I don't know" when asked about wrongness and justice, after observing punishment (Fig S6.2C). To help clarify why an authority might punish an act, despite prior information that the act is harmless, in Version 2 (pre-registration: https://osf.io/kngu9) we added one additional sentence after introducing Paji: "Leaders in this society vary in how much they care about justice and treating people fairly, and whether they have biases towards some people". This additional sentence reduced the number of "I don't know" responses, so the results of Version 2 are presented in the main text. Nevertheless, all key results were similar in the two versions (Fig S6.2).

In study 4, we included two comprehension checks (3-choice questions) before measuring the prior beliefs, asking: 1) Who has power and influence in this society?, and 2) How harmful is daxing? The correct responses were "Paji" (from Paji, Fadi, I don't know options) in all conditions, and "very harmful", "not-harmful" and "I don't know" in the "Wrong", "Not-wrong" and "Just" conditions, respectively. We only analyzed the data from participants who responded to both questions correctly (excluded 24 participants out of 180 in version 1, and 16 participants out of 181 in version 2).

Polarization simulations. We simulated the evolution of beliefs in three observers of the same sequence of punitive choices (i.e., 4 harsh punishments), who differed in their prior beliefs. The three observers' prior beliefs were matched to the average measured prior beliefs in the "Wrong", "Not-wrong" and "Just" conditions in study 4 (version 2). In study 4, we did not measure participants' inferences about the authority's bias and selfishness; we thus used the average prior beliefs from the corresponding prior conditions in study 1. For simulating the evolution of beliefs, we used these priors as initial input to the inverse planning model that was fit in study 1, as explained in the main text.

Supplementary results.

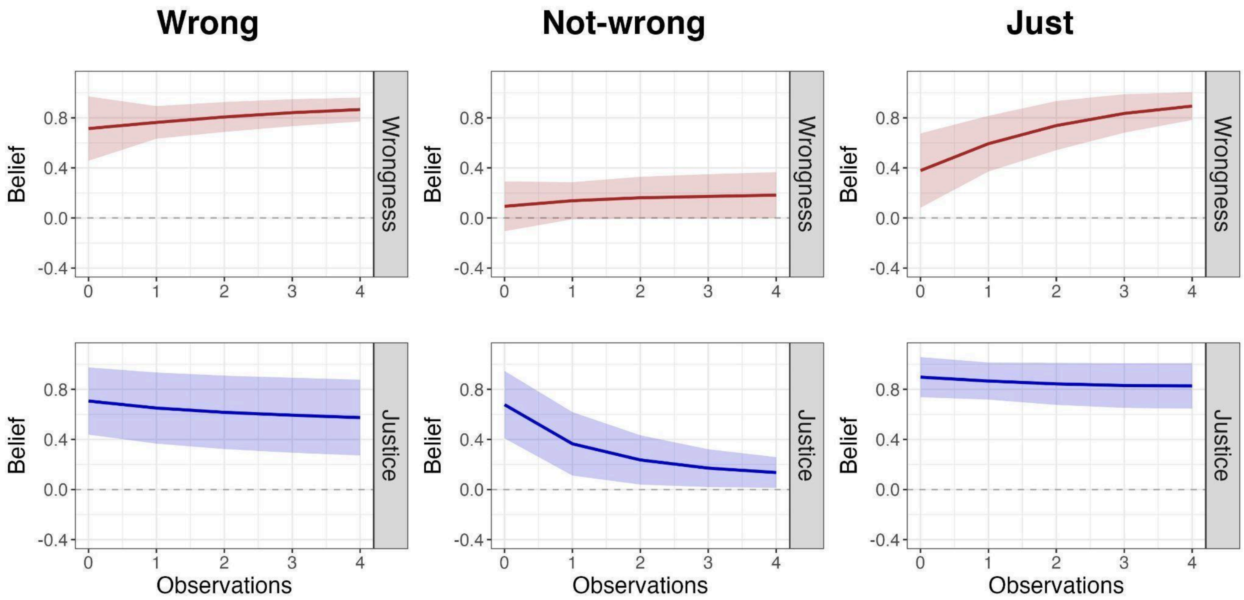

Fig. S6.1 Simulated belief evolution in each prior condition The simulations used the inverse planning model fit in study 1 and prior parameters measured in study 4 (i.e., observation=0) to make predictions about the evolution of beliefs after receiving shared evidence by all three observers from the "Wrong", "Not-wrong" and "Just" groups. The observations consist of 4 consecutive harsh punishments of 'daxing'.

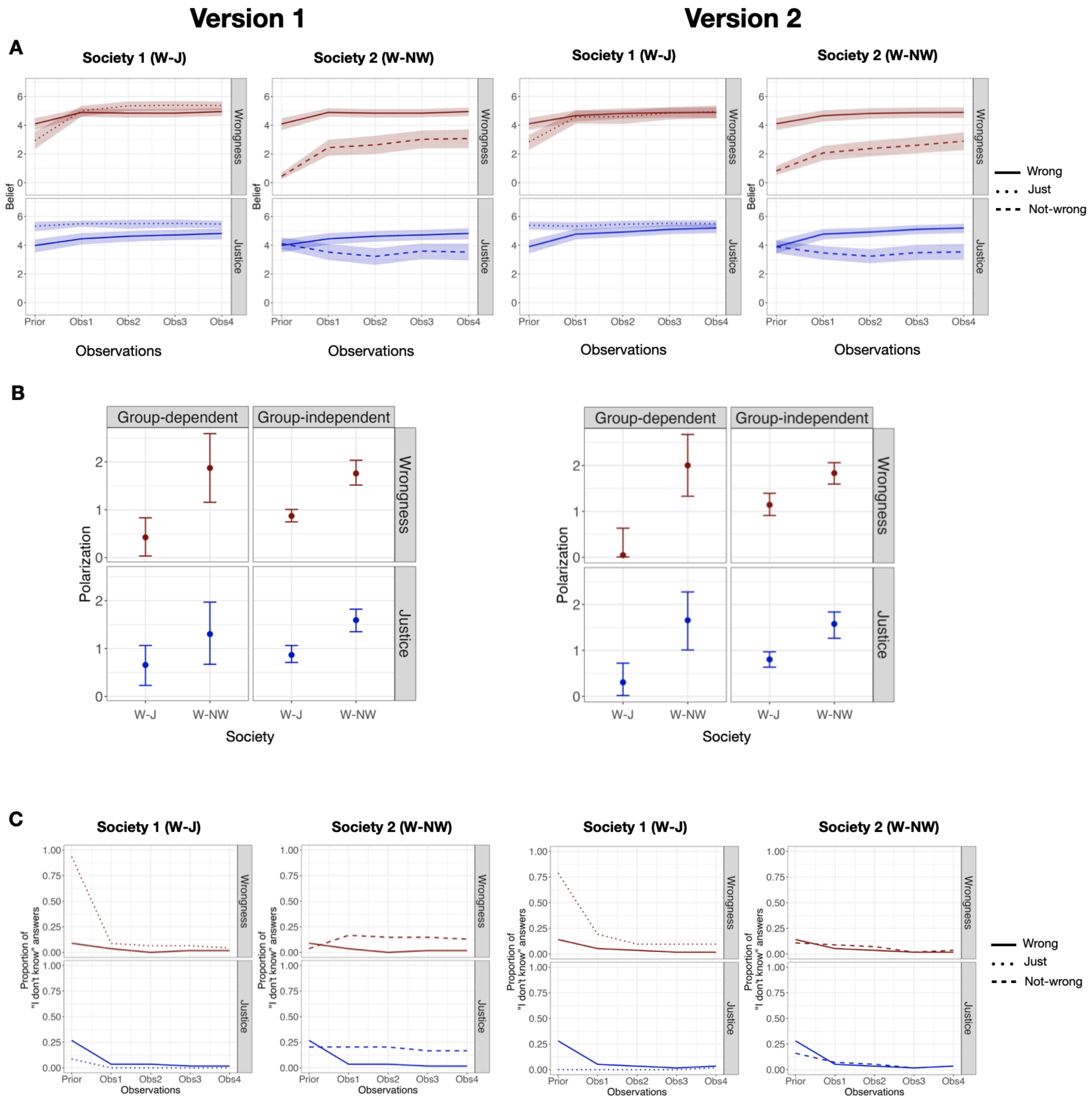
Version 1
Version 2
A
Society 1 (W-J)
Society 2 (W-NW)
Belief
Wrongness
Justice
Prior
Obs1
Obs2
Obs3
Obs4
Observations
Wrong
Just
Not-wrong
B
Group-dependent
Group-independent
Polarization
W-J
W-NW
Society
C
Proportion of "I don't know" answers

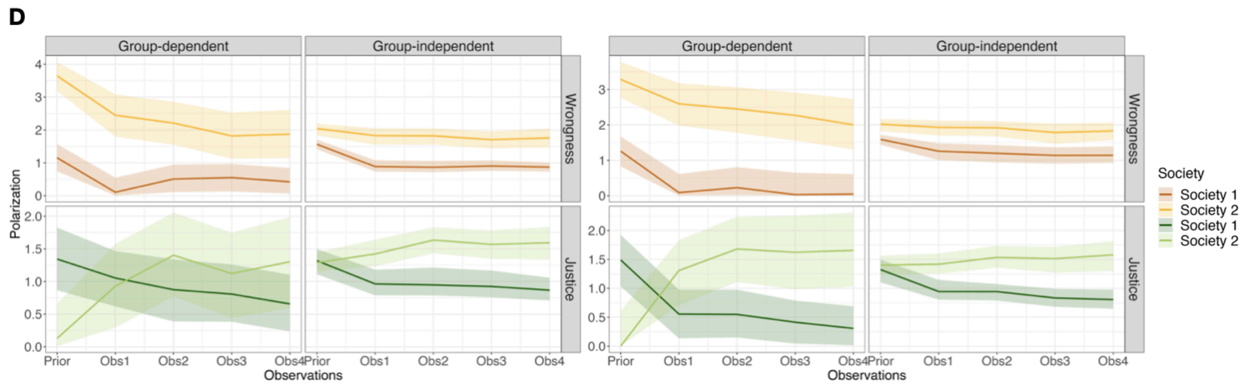

Fig. S6.2 Study 4 results are similar for versions 1 and 2. Society 1 consists of individuals in the “Wrong” and “Just” condition, and society 2 of “Wrong” and “Not-wrong” conditions. A) evolution of beliefs for the two groups within each society. The shaded ribbon shows the bootstrapped 95% CI of the mean, where “I don’t know” responses are replaced by a uniform distribution over the scale; B) The group-dependent measure is the difference between the two group means, and the group-independent measure is the average absolute deviation from the mean. Therefore, both measures are in the same units as the belief measures. The zero value indicates no difference between the means of two groups for the group-dependent measure, and a delta distribution for the group-independent measure. The error bars show the bootstrapped 95% CI; C) evolution of proportion of “I don’t know. All values are equally” responses within each prior condition, plotted for each society separately; D) evolution of polarization measures within each society.

Pre-registered analyses for the descriptive statistics of beliefs evolution

<u>W-L society:</u>

For beliefs about wrongness, there was a significant effect of condition (“Just” vs “Wrong”: β = -0.847, SE = 0.214, $t(541) = -3.96$, $p = 8.4e-05$), a significant positive effect of number of observed punishments (β = 0.179, SE = 0.060, $t(541) = 2.97$, $p = 0.0031$), and a significant positive interaction between condition and number of observations (β = 0.267, SE = 0.087, $t(541) = 3.06$, $p = 0.0023$).

For beliefs about justice, there was a significant effect of condition (“Just” vs “Wrong”: β = 1.164, SE = 0.166, $t(541) = 7.01$, $p = 6.97e-12$), a significant positive effect of number of observed punishments (β = 0.293, SE = 0.047, $t(541) = 6.26$, $p = 7.91e-10$), and a significant negative interaction between condition and number of observations (β = -0.251, SE = 0.068, $t(541) = -3.70$, $p = 0.00024$).

<u>W-NW society:</u>

For beliefs about wrongness, there was a significant effect of condition (“Not-wrong” vs “Wrong”: β = -3.098, SE = 0.246, $t(561) = -12.58$, $p < 2e-16$), a significant positive effect of

number of observed punishments (β = 0.179, SE = 0.071, $t(561) = 2.53$, $p = 0.012$), and a significant positive interaction between condition and number of observations (β = 0.289, SE = 0.101, $t(561) = 2.87$, $p = 0.0042$).
For beliefs about justice, there was a significant effect of condition ("Not-wrong" vs "Wrong": β = -0.529, SE = 0.230, $t(561) = -2.30$, $p = 0.022$), a significant positive effect of number of observed punishments (β = 0.293, SE = 0.066, $t(561) = 4.43$, $p = 1.16e-05$), and a significant negative interaction between condition and number of observations (β = -0.363, SE = 0.094, $t(561) = -3.86$, $p = 0.00013$).

Pre-registered analyses for the patterns of polarization

Beliefs about wrongness

The group-dependent polarization (between-group divergence) showed a significant main effect of society (Society 2 vs Society 1: β = 2.270, SE = 0.339, $t(6) = 6.70$, $p = 5.38e-04$), a significant negative main effect of number of observations (β = -0.248, SE = 0.098, $t(6) = -2.54$, $p = 0.044$), but no significant interaction between society and observations (β = -0.041, SE = 0.138, $t(6) = -0.29$, $p = 0.778$).
The group-independent polarization (dispersion) also revealed a significant main effect of society (Society 2 vs Society 1: β = 0.533, SE = 0.094, $t(6) = 5.65$, $p = 0.0013$), a significant negative main effect of observations (β = -0.102, SE = 0.027, $t(6) = -3.73$, $p = 0.010$), but no significant interaction between society and observations (β = 0.049, SE = 0.039, $t(6) = 1.28$, $p = 0.247$).

Beliefs about justice

The group-dependent polarization (between-group divergence) revealed no significant main effects of society (Society 2 vs Society 1: β = -0.636, SE = 0.448, $t(6) = -1.42$, $p = 0.206$) or observations (β = -0.251, SE = 0.129, $t(6) = -1.94$, $p = 0.101$), but a significant positive interaction between society and number of observations (β = 0.613, SE = 0.183, $t(6) = 3.35$, $p = 0.015$).
The group-independent polarization (dispersion) showed a marginally significant main effect of society (Society 2 vs Society 1: β = 0.199, SE = 0.093, $t(6) = 2.13$, $p = 0.077$), a significant negative main effect of observations (β = -0.115, SE = 0.027, $t(6) = -4.25$, $p = 0.0054$), and a significant positive interaction between society and number of observations (β = 0.160, SE = 0.038, $t(6) = 4.19$, $p = 0.0057$).

7. Individual differences

Polarization simulations for RWA. We simulated the evolution of beliefs in two observers of the same sequence of punitive choices (i.e., 5 harsh punishments), who differed in their prior beliefs. The two observers' prior beliefs were matched to the average measured prior beliefs for individuals with high and low RWA (median split), within each of the 6 scenarios in the "Somewhat-wrong" condition in study 1. For simulations, we used the inverse planning model that was fit in study 1, as explained in the main text.

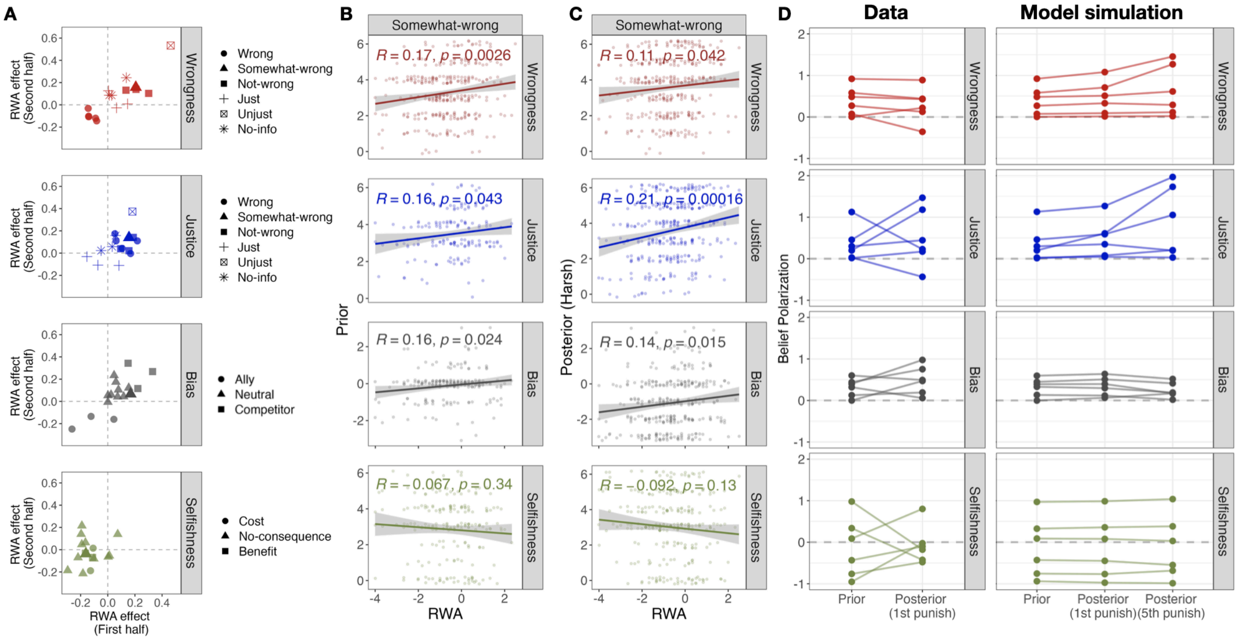

Fig. S7.1 Individual differences in prior beliefs and interpretations of punishment. A) Reliability of the effect of RWA on self-reported prior beliefs within each prior condition, combined across all three studies. The x and y-axes show RWA effects measured in independent subsets of the scenarios (1-3 vs 4-6). Point shape indicates prior condition. B, C) Correlation between RWA and self-reported prior and posterior beliefs after observing harsh punishment, in the "Somewhat-wrong" condition. D) The difference between (averaged) prior and posterior beliefs of people with high versus low RWA (i.e., median split), within each of the 6 scenarios in the "Somewhat-wrong" condition (i.e., the group-dependent polarization where groups are defined as people with high vs low RWA). The left plot shows the experimental data. The right plot shows the model simulations, where the high and low RWA observers start from the empirically measured prior beliefs, and the model is used to simulate the evolution of their beliefs after observing 1 and 5 instances of harsh punishment by the authority in response to a target act, within each of the 6 scenarios.

Supplementary results.

Across our three studies, before observing any punitive response, people higher in RWA judged the authority as more motivated by justice (in study 1 across all conditions, β=0.133, std=0.049, t(375.5)=2.73, p=0.0066) and more impartial (*NP) (in study 2 across "Ally" conditions, β=-0.200, std=0.051, t(501.5)=-3.90, p=1.08e-04; "Competitor" conditions: β =0.295, std=0.059, t(489)=5.03, p=6.92e-07), and the target act as more wrong (in study 1 across all conditions, β=0.233, std=0.046, t(444)=5.01, p=7.87e-07), even when given the exact same prior information (Fig S7.1A, see effect of RWA within each prior condition). The individual differences in prior beliefs are particularly pronounced when the contextual information suggests that punishment would be illegitimate (Fig S7.1A, "Somewhat-wrong", "Not-wrong", "Unjust", "Ally" and "Competitor" conditions)

Apart from the effect of RWA on prior belief values, we found an effect of RWA on the uncertainty of prior beliefs as well. In a pre-registered analysis, we found that overall, those higher on RWA were a priori more certain in their beliefs about wrongness of the act and the authority's motives, indicated by a lower likelihood of answering "I don't know" provided the same information (main effect of RWA in study 1, on wrongness: β=-0.452, std=0.155, z=-2.91, p=0.0036; on justice: β=-0.617, std=0.152, z=-4.07, p=4.73e-05). We did not consider this effect in our belief polarization simulations.
